# The plastid-encoded PsaI subunit stabilizes photosystem I during leaf senescence in tobacco

**DOI:** 10.1093/jxb/erx009

**Published:** 2017-02-09

**Authors:** Mark Aurel Schöttler, Wolfram Thiele, Karolina Belkius, Sonja Verena Bergner, Claudia Flügel, Gal Wittenberg, Shreya Agrawal, Sandra Stegemann, Stephanie Ruf, Ralph Bock

**Affiliations:** 1 Max Planck Institute of Molecular Plant Physiology, Am Mühlenberg 1, 14476 Potsdam-Golm, Germany

**Keywords:** Chloroplast transformation, heat stress, leaf senescence, light acclimation, photosynthesis, photosystem I, plastocyanin, PsaI, tobacco.

## Abstract

PsaI is the only subunit of PSI whose precise physiological function has not yet been elucidated in higher plants. While PsaI is involved in PSI trimerization in cyanobacteria, trimerization was lost during the evolution of the eukaryotic PSI, and the entire PsaI side of PSI underwent major structural remodelling to allow for binding of light harvesting complex II antenna proteins during state transitions. Here, we have generated a tobacco (*Nicotiana tabacum*) knockout mutant of the plastid-encoded *psaI* gene. We show that PsaI is not required for the redox reactions of PSI. Neither plastocyanin oxidation nor the processes at the PSI acceptor side are impaired in the mutant, and both linear and cyclic electron flux rates are unaltered. The PSI antenna cross section is unaffected, state transitions function normally, and binding of other PSI subunits to the reaction centre is not compromised. Under a wide range of growth conditions, the mutants are phenotypically and physiologically indistinguishable from wild-type tobacco. However, in response to high-light and chilling stress, and especially during leaf senescence, PSI content is reduced in the mutants, indicating that the I-subunit plays a role in stabilizing PSI complexes.

## Teaser

In higher plants, the plastome-encoded photosystem I-subunit PsaI is specifically required for photosystem I stability during high-light and chilling stress and leaf senescence.

## Introduction

PSI catalyses the final step of linear photosynthetic electron transport, oxidizing plastocyanin and reducing ferredoxin and, via the ferredoxin NADP^+^ oxidoreductase, ultimately NADP^+^ in a light-driven reaction sequence. In parallel, linear electron transport also generates a proton motive force (pmf) across the thylakoid membrane, which drives ATP synthesis by the chloroplast ATP synthase. In addition to NADPH production, electrons on the PSI acceptor side can be partitioned into other reactions, most notably cyclic electron transport around PSI, which generates an extra pmf to produce ATP without net NADPH production (recently reviewed by Yamori and Shikanai, 2016; Finazzi and Johnson, 2016).

In higher plants, PSI is composed of 14 subunits (PsaA to PSAL, PSAN, and PSAO), with an additional complement of four different light harvesting complex A (LHCA) antenna proteins, which are stably bound to one side of PSI and form a half-moon-shaped belt (reviewed by Jensen *et al.*, 2007; Schöttler *et al.*, 2011). The weakly expressed LHCA5 and LHCA6 proteins are present in sub-stoichiometric amounts relative to PSI (Ganeteg *et al.*, 2004), and could play a role in supercomplex formation between PSI and the NDH complex to facilitate cyclic electron transport (Peng *et al.*, 2009; Kouřil *et al.*, 2014). All redox-active cofactors of PSI are bound to the three plastid genome-encoded subunits PsaA–C. Loss of any of these three subunits abolishes PSI accumulation (Takahashi *et al.*, 1991; Redding *et al.*, 1999). PsaA and B bind the redox-active chlorophyll-a dimer P_700_, where the light-induced charge separation occurs, as well as the subsequent components of the intra-complex electron transport chain, such as the first non-chlorophyll electron acceptor A_1_, a phylloquinone, and F_X_, the first iron-sulfur (FeS) cluster on the PSI acceptor side. The other FeS clusters, F_A_ and F_B_, are bound to PsaC, which is located on the stromal side of PSI. The stable binding of PsaC requires the nuclear-encoded subunits PSAD and PSAE, which constitute the stromal ridge involved in ferredoxin binding. Knocking out PSAD or PSAE severely affects PSI function; in *Arabidopsis thaliana*, the D-subunit is essential for PSI stability and photoautotrophic growth (Haldrup *et al.*, 2003, Ihnatowicz *et al.*, 2004).

P_700_^+^ reduction via plastocyanin is supported by PSAF and the luminal N-subunit (Haldrup *et al.*, 1999). PSAF-deficient *A. thaliana* mutants also suffer from a strongly decreased PSI accumulation, which abolishes their photoautotrophic growth (Haldrup *et al.*, 2000). The other subunits of PSI do not play comparably important roles, and their knockout does not abolish photoautotrophic growth. Often, PSI accumulation is slightly reduced, indicating a minor role in the stability of PSI. In many cases, however, the antenna cross section per PSI reaction centre is altered. This is especially the case when accumulation of the subunits interacting with the LHCA belt is compromised, such as in a plastid-transformed (transplastomic) mutant deficient in the small plastid-encoded J-subunit (Schöttler *et al.*, 2007a), but also in mutants lacking PSAK (Jensen *et al.*, 2000). Interestingly, despite its location close to the LHCI belt, the loss of the K-subunit was also shown to impair enlargement of the PSI antenna cross section by state transitions (Varotto *et al.*, 2002). State transitions balance the excitation rates of the photosystems in low light by a phosphorylation-induced redistribution of LHCII from PSII to PSI (reviewed by Goldschmidt-Clermont and Bassi, 2015). When PSII excitation exceeds that of PSI, there is an accumulation of reduced plastoquinol, which binds to the cytochrome b_6_f complex (b_6_f) and activates the thylakoid kinase STN7. STN7 phosphorylates trimeric LHCII, which then detaches from PSII and migrates towards PSI in the grana margins, thus increasing the PSI antenna cross section. PSI binds the free LHCII trimers via the subunits PSI-H (Lunde *et al.*, 2000), PSI-O (Jensen *et al.*, 2004), and PSI-L (Lunde *et al.*, 2003). In *PSAH* knockout mutants, the PSAL subunit is depleted to approximately 10–30% of the wild-type level (Lunde *et al.*, 2000; Varotto *et al.*, 2002), suggesting that their state transitions defect might be indirectly conferred by the loss of PSAL. The H- and L-subunits together with the I-subunit constitute a nose-shaped structure on the PSI side opposite to the LHCA belt. In addition to its function in state transitions, new crystallographic data implicate that the H-subunit is involved in the formation of the plastocyanin-binding site (Mazor *et al.*, 2015). It has also been suggested that the H-subunit interacts with the stromal ridge (Qin *et al.*, 2015).

The only subunit whose precise physiological function has not yet been addressed in photosynthetic eukaryotes is the small I-subunit, which forms a single transmembrane α-helix. In cyanobacteria, PsaI has been implicated in PSI trimerization (Xu *et al.*, 1995), most likely via stabilization of the subunits PsaL and PsaM, which mediate trimer formation (Schluchter *et al.*, 1996). No further effects of a *psaI* disruption on PSI content, PSI activity, or growth behaviour have been observed (Nakamoto, 1995). After endosymbiosis, the PSI monomer became the functional unit owing to the evolution of membrane-intrinsic LHCs that replaced the phycobilisomes of cyanobacteria as antenna systems. While PsaM was lost, the I-subunit was retained by photosynthetic eukaryotes. This indicates that the I-subunit either has an additional, conserved function not related to trimerization both in cyanobacteria and eukaryotes, or that it has acquired a novel role in higher plants. Crystallographic data of pea (*Pisum sativum*) PSI support a structural role for the I-subunit; it coordinates four carotenoids (Amunts *et al.*, 2010), which might function in the protection of PSI against reactive oxygen species (ROS). Furthermore, PsaI physically interacts with the PsaB reaction centre subunit and the small peripheral subunits PSAL and PSAH (Amunts *et al.*, 2010). Therefore, the stable binding of PSAH and PSAL might be impaired in a PsaI mutant (Amunts *et al.*, 2010; Mazor *et al.*, 2015; Qin *et al.*, 2015). This should then result in an indirect state transition defect. Because of the putative role of the H-subunit in plastocyanin oxidation (Mazor *et al.*, 2015) and its interaction with the stromal ridge subunit PSAD (Mazor *et al.*, 2015; Qin *et al.*, 2015), redox reactions in PSI might also be indirectly affected by a *psaI* knockout.

A recent study of PSI from a transplastomic tobacco (*Nicotiana tabacum*) ∆*psaI* mutant suggests that the I-subunit is, indeed, required for stable binding of PSAH and PSAL and stabilization of the LHCII docking site of PSI (Plöchinger *et al.*, 2016). Surprisingly, despite the slightly slower growth of their ∆*psaI* mutant under light-limited conditions of 125 µE m^−2^ s^−1^, the authors could not detect any *in vivo* defects in state transitions or PSI antenna function in their ∆*psaI* mutant. The mutant was indistinguishable from wild-type tobacco in all photosynthetic parameters reported. The authors did observe a rapid LHCII phosphorylation in darkness after illumination with far-red light, but that response was also detected in other PSI mutants of tobacco and Arabidopsis and, therefore, does not represent a specific functional defect of the ∆*psaI* mutant. The authors did not investigate if the loss of PsaI affects photosynthesis and plant growth under different growth conditions and stresses. They also did not assess a possible effect of the *psaI* knockout on cyclic electron flux.

Here, we report the generation of transplastomic tobacco mutants in which the *psaI* gene was disrupted. We identified three conditions in which loss of the I-subunit resulted in impaired PSI accumulation. Upon high-light and chilling stress and, especially, during leaf senescence, PSI contents were specifically reduced in the mutants, indicating that the I-subunit plays a role in stabilizing PSI and protecting it from oxidative damage and proteolytic degradation.

## Material and methods

### Plant cultivation

Seeds were germinated under long-day conditions (16 h light) at a light intensity of 100 µE m^−2^ s^−1^. The day temperature was 22°C, the relative humidity 75%. During the night, temperature and relative humidity were decreased to 18°C and 70%, respectively. Four weeks after germination, plants were either maintained at 100 µE m^−2^ s^−1^ or transferred to other light regimes (intermediate: 350 µE m^−2^ s^−1^; high light: 1000 µE m^−2^ s^−1^). All other environmental parameters were kept constant, except for the heat and chilling experiments, in which the temperature was increased to 38°C during the day and to 34°C during the night, or decreased to 12°C during the day and 8°C during the night, respectively. After a minimum time of 14 days to allow full acclimation to the new environment, the first leaf pair to develop under the respective growth conditions was used for all measurements.

### Vector construction and chloroplast transformation

The region containing the plastid *psaI* gene was cloned from tobacco chloroplast DNA as a 2414-bp BamHI/HbaI fragment into vector pBluescript SK+. A chimeric *aadA* gene cassette (Svab and Maliga, 1993) was inserted as a blunt-end fragment into a unique StuI site within *psaI*, thus disrupting the reading frame. A clone containing the *aadA* cassette in the same transcriptional orientation as *psaI* was selected as the plastid transformation vector.

Plastid transformation experiments were carried out using the biolistic protocol (Svab and Maliga, 1993). Young leaves from tobacco plants grown under aseptic conditions on Murashige and Skoog (MS) medium were bombarded with plasmid DNA-coated 0.6-µm gold particles using the PDS-1000/He biolistic gun with the Hepta Adaptor (BioRad). Primary spectinomycin-resistant lines were selected on an MS-based plant regeneration medium containing 500 mg L^−1^ spectinomycin (Svab and Maliga, 1993). Spontaneous antibiotic-resistant mutants were eliminated by growth assays on regeneration medium containing both spectinomycin and streptomycin (500 mg L^−1^ each; Bock, 2001). Several independent transplastomic lines were generated and subjected to three additional rounds of regeneration on spectinomycin-containing medium to obtain homoplasmic tissue. Regenerated homoplasmic plantlets were rooted on hormone-free MS medium and then transferred to soil and grown to maturity under standard greenhouse conditions.

### Isolation of nucleic acids and gel blot analyses

Total plant DNA was extracted from leaf samples using a cetyltrimethylammoniumbromide (CTAB)-based method (Doyle and Doyle, 1990). Total RNA was isolated using the peqGOLD TriFast reagent (Peqlab GmbH, Erlangen) following the manufacturer’s protocol.

For Southern blot analysis, 5 μg samples of DNA were digested with XhoI and HindIII, and separated by gel electrophoresis in 1% agarose gels. For northern blot analyses, RNA samples were electrophoresed in formaldehyde-containing 1% agarose gels. Gel electrophoretically separated nucleic acids were transferred onto Hybond XL membranes (GE Healthcare) by capillary blotting. Hybridization probes were purified by agarose gel electrophoresis followed by extraction of the DNA fragments of interest from excised gel slices. Probes were labelled with α[^32^P]dCTP by random priming using a multiprime DNA labelling system (GE Healthcare). Hybridizations were performed at 65°C using standard procedures.

Restriction fragments or PCR products generated by amplification with gene-specific primers were used as hybridization probes. A 420-bp EcoRV restriction fragment covering the *ycf4* coding region was used as the restriction fragment length polymorphism (RFLP) probe to verify plastid transformation and assess the homoplasmic state of the transformants. To detect transcripts in northern blot experiments, the following gene-specific probes were amplified by PCR: *psaI* (with primers 5′-CCCTTCTATGACAAATTTGA-3′ and 5′-CCTTCCCCTGTTCCCGCCTACG-3′), *ycf10* (with primers 5′-ATGGCAAAAAAGAAAGCATTCACTC-3′ and 5′-CCAATCATTAATTCCCAACCGTG-3′), and *petA* (with primers 5′-GCACAGCAGGGTTATGAAAATCC -3′ and 5′-CGCCCTCGGAAACAAGAAGTTCTG-3′). To detect *ycf4* transcripts, the same probe as for the RFLP analysis was used.

### Gas exchange measurements

Gas exchange measurements were performed with a GFS-3000 open gas exchange system equipped with the LED array unit 3055-FL as an actinic light source. Light-response curves of CO_2_ assimilation were measured at 22°C cuvette temperature with 17500 ppm humidity and a saturating CO_2_ concentration of 2000 ppm. After leaf respiration was determined in darkness, the actinic light intensity was stepwise increased to 2000 µE m^−2^ s^−1^. Gas exchange was recorded at each light intensity until a steady state of transpiration and leaf assimilation was reached. The quantum efficiency of CO_2_ fixation was calculated in the linear light-response range between 0 and 100 µE m^−2^ s^−1^.

### ATP synthase activity and light-induced pmf

The thylakoid membrane conductivity for protons (gH^+^) was used as a measure for ATP synthase activity. It was determined on intact leaves from the decay kinetics of the electrochromic shift signal (ECS) during a short interval of darkness. The experiments were performed at 22°C. Leaves were pre-illuminated for 5 min with saturating light (1400 µE m^−2^ s^−1^) so that photosynthesis was fully activated and ATP synthase activity was not limited by ATP consumption by the Calvin–Benson cycle. The saturating illumination was interrupted by 15-s intervals of darkness, and the rapid first phase of the decay kinetic of the electrochromic shift during the first 250 ms of darkness was fitted with a single exponential decay function. The reciprocal value of the time constant was used as a measure of ATP synthase activity. Signals were measured and deconvoluted using a KLAS-100 spectrophotometer (Heinz Walz GmbH, Effeltrich, Germany) as previously described (Rott *et al.*, 2011). The maximum amplitude of the ECS during the first phase of its relaxation kinetic was also used as a measure for the total light-induced pmf across the thylakoid membrane (ECS_t_). To take differences in leaf chlorophyll content into account, the signal amplitude was normalized to the chlorophyll content per leaf area (Rott *et al.*, 2011; Davis *et al.*, 2016).

### Cytochrome f redox kinetics

Leaves were pre-illuminated as described for the ATP synthase measurements to fully activate the Calvin–Benson cycle and avoid an acceptor-side limitation of PSI. Cytochrome f was fully oxidized by a saturating light pulse, followed immediately by a short interval of darkness, to allow its complete reduction. The fully reduced state of cytochrome f was reached after a maximum of 500 ms in darkness, and the amplitude of the difference transmission signal between the fully oxidized state during the light pulse and the fully reduced state in darkness was used as a measure of redox-active cytochrome f. The cytochrome f signal was measured with the KLAS-100 spectrophotometer and deconvoluted as previously described (Klughammer *et al.* 1990; Rott *et al.* 2011). The deconvoluted difference transmission signal was normalized to the chlorophyll content of the leaf.

### Chlorophyll-a fluorescence measurements and leaf absorptance

A F-6500 fluorometer (Jasco Inc., Groß-Umstadt, Germany) was used to measure 77 K chlorophyll-a fluorescence emission spectra on freshly isolated thylakoid membranes equivalent to 10 µg chlorophyll mL^−1^. The sample was excited at 430-nm wavelength with a bandwidth of 10 nm, and the emission spectrum was recorded between 655- and 800-nm wavelengths in 0.5-nm intervals with a bandwidth of 1 nm. *In vivo* measurements of chlorophyll-a fluorescence parameters at 22°C were performed using a Dual-PAM instrument (Heinz Walz GmbH). Light-response curves of linear electron flux, non-photochemical quenching (qN; Krause and Weis, 1991), and the redox state of the PSII acceptor side (qL; Kramer *et al.*, 2004) were measured after 30 min of dark adaptation. The light intensity was increased stepwise from 0 to 2500 µE m^−2^ s^−1^, with a measuring time of 150 s for each light intensity under light-limited conditions and of 60 s under light-saturated conditions. Linear electron transport was corrected for leaf absorptance, which was calculated from leaf transmittance and reflectance spectra as 100% minus transmittance (%) minus reflectance (%). Spectra were measured between 400- and 700-nm wavelengths using an integrating sphere attached to a photometer (V650, Jasco Inc.). The spectral bandwidth was set to 1 nm, and the scanning speed was 200 nm min^−1^.

### Thylakoid membrane isolation and photosynthetic complex quantification

Thylakoid membranes were isolated as described previously (Schöttler *et al.*, 2004). The chlorophyll content and a/b ratio were determined in 80% (v/v) acetone according to Porra *et al.* (1989). The contents of PSII and the b_6_f were determined from difference absorbance signals of cytochrome b_559_ (PSII) and the cytochromes b_6_ and f in destacked thylakoids equivalent to 50 µg chlorophyll mL^−1^ (Kirchhoff *et al.*, 2002). All cytochromes were fully oxidized by the addition of 1 mM potassium hexacyanoferrate (III), and then stepwise reduced by the addition of 10 mM sodium ascorbate to reduce the high-potential form of cytochrome b_559_ and cytochrome f, and the addition of 10 mM sodium dithionite to reduce the low potential form of cytochrome b_559_ and the two b-type hemes of cytochrome b_6_. Using a V-550 spectrophotometer equipped with a head-on photomultiplier (Jasco GmbH) at each of the three redox potentials, absorbance spectra were measured between 575- and 540-nm wavelengths. The spectral bandwidth was 1 nm and the scanning speed 100 nm min^−1^. Ten spectra were averaged per redox condition. Difference spectra were calculated by subtracting the spectrum measured in the presence of hexacyanoferrate from the ascorbate spectrum, and by subtracting the ascorbate spectrum from the spectrum measured in the presence of dithionite, respectively. Finally, a baseline calculated at wavelengths between 540 and 575 nm was subtracted from the signals. Then, the difference spectra were deconvoluted using reference spectra as previously described (Kirchhoff *et al.*, 2002; Lamkemeyer *et al.*, 2006).

PSI was quantified from light-induced difference absorbance changes of P_700_. Thylakoids equivalent to 50 µg chlorophyll mL^−1^ were solubilized in the presence of 0.2% (w/v) n-dodecyl-β-D-maltoside (DDM). After the addition of 10 mM sodium ascorbate as the electron donor and 100 µM methylviologen as the electron acceptor, P_700_ photo-oxidation was achieved by applying a light pulse of 250 ms (2000 µmol photons m^−2^ s^−1^). Measurements were performed with the plastocyanin-P_700_ version of the Dual-PAM instrument (Heinz Walz GmbH). Plastocyanin contents, relative to PSI, were determined in intact leaves and then recalculated based on the absolute PSI quantification performed in isolated thylakoids (Schöttler *et al.*, 2007a). The same instrument was also used to determine light-response curves of the acceptor-side limitation and donor-side limitation of PSI in intact leaves, which were calculated according to Klughammer and Schreiber (2016).

### Sucrose density gradient ultracentrifugation of isolated thylakoids

To separate photosynthetic multiprotein complexes by sucrose density gradient ultracentrifugation (SDG), isolated thylakoid membranes corresponding to 560 µg chlorophyll were centrifuged for 5 min (4500*g*, 4°C), washed with 10 mM HEPES (pH 7.5) and 5 mM EDTA (pH 8.0), and resuspended in 10 mM HEPES (pH 7.5) at a concentration of 0.8 mg chlorophyll mL^−1^. Solubilization of thylakoids was achieved by adding 0.9% (v/v) DDM and incubating for 20 min on ice with regular mixing every few minutes. Samples were centrifuged for 10 min (14000*g*, 4°C) and the supernatant was loaded onto discontinuous SDGs (sucrose 0.4–1 M in 0.05% DDM, 5 mM Tricine pH 8.0) prior to separation by ultracentrifugation with an SW41Ti rotor (Beckmann) at 4°C (33000 rpm, 20 h). SDG fractions were withdrawn from the bottom to the top of the centrifugation tube using the fractionation device Auto Densi-Flow (Labconco), and chlorophyll content and chlorophyll a/b ratio of the obtained fractions were determined. To separate the protein complexes by SDS-PAGE followed by immunodetection, 30 µL of each fraction (5–30) from wild-type and *ΔpsaI* gradients were used.

### Protein gel electrophoresis and immunoblotting

Thylakoid proteins were separated by SDS-PAGE and then transferred to a polyvinylidene membrane (Hybond P, GE Healthcare) using a tank blot system (Perfect Blue Web M, VWR International GmbH, Darmstadt, Germany). Immunochemical detection was performed using an enhanced chemiluminescence detection reagent (ECL Prime, GE Healthcare) according to the manufacturer’s instructions. Chemiluminescence was detected using the G:BOX XT4 imaging system (Syngene, Cambridge, UK). The following specific antibodies against the photosynthetic proteins were purchased from Agrisera AB (Vennäs, Sveden): PsbB (order number: AS04 038), PsbD (AS 06 146), LHCB1, LHCB4 (AS04 045), PetA (AS06 119), PetB (AS03 034), PETC (AS08 330), PsaA (AS06 172), PsaB (AS10 695), PSAD (AS09 461), PSAF (AS06 104), PSAH (AS06 105), PSAK (AS04 049), PSAL (AS06 108), PSAN (AS06 109), LHCA1 (AS01 005), LHCA2 (AS01 006), LHCA3 (AS01 007), LHCA4 (AS01 008), and AtpB (AS05 085). Antibodies against Ycf4 and Y3IP1 were produced by Krech *et al.* (2012). To determine protein oxidation levels, the Millipore Oxyblot^TM^ protein oxidation detection kit (Merck Chemicals GmbH, Darmstadt, Germany) was used according to the manufacturer’s protocol. Thylakoid proteins equivalent to 2 µg chlorophyll were loaded per reaction.

## Results

### Targeted inactivation of the *psaI* gene in the tobacco chloroplast genome

**Fig. 1.**
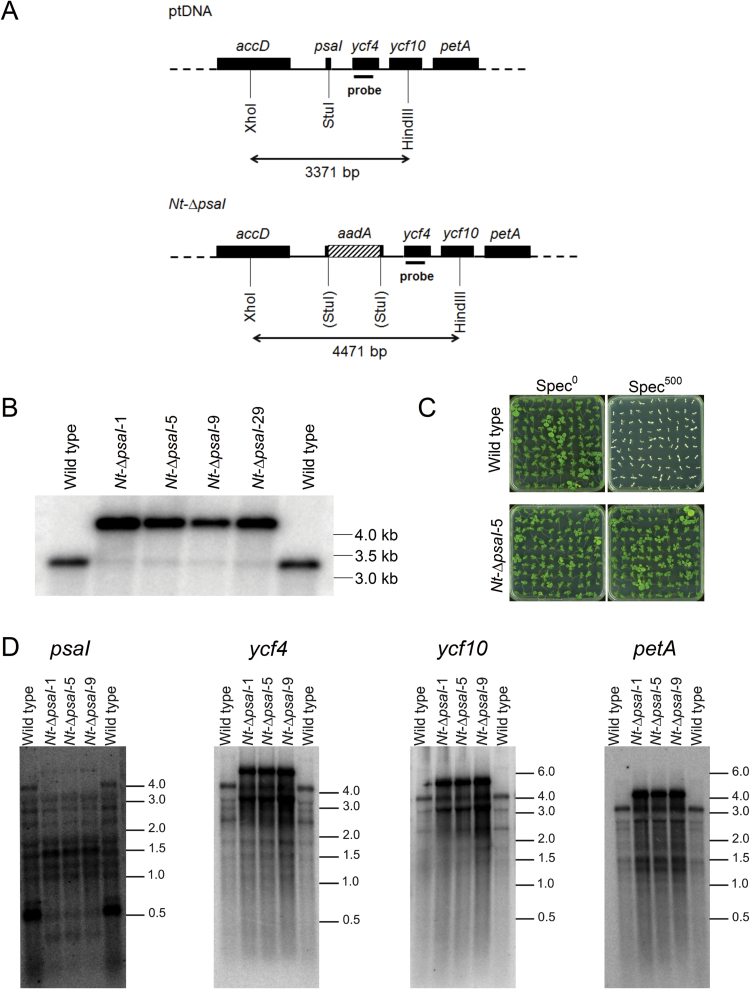
Generation of transplastomic tobacco plants with a disrupted *psaI* gene. (**A**) Physical maps of the wild-type plastid genome (plastome) and the transformed plastome harbouring the selectable marker gene *aadA* to disrupt the *psaI* gene (*Nt*-∆*psaI*). (**B**) Confirmation of plastid transformation and integration of the *aadA* marker via homologous recombination by Southern blotting. Total DNA was digested with the restriction enzymes XhoI and HindIII, generating a restriction fragment of 3371 bp in the wild type and a fragment of 4471 bp in four representative transplastomic *psaI* knockout lines. (**C**) Confirmation of homoplasmy of the transplastomic plants by seed assays on antibiotic-free and antibiotic-containing media. Only one example line (*Nt*-∆*psaI*-5) is shown. See text for details. (**D**) Northern blot analysis to examine the expression of *psaI* and the three other genes in the operon.

The *psaI* gene is the first cistron of a polycistronic transcription unit in the plastid genome that also comprises *ycf4*, *ycf10*, and the b_6_f subunit gene *petA*. Ycf4 is an auxiliary protein required for PSI assembly (Krech *et al.*, 2012), and Ycf10 is a non-essential inner envelope protein possibly involved in inorganic carbon uptake into the chloroplast (Rolland *et al.*, 1997). To knockout *psaI*, the gene was disrupted by inserting a selectable marker gene cassette (the spectinomycin-resistance gene *aadA*; Svab and Maliga, 1993). To minimize disturbance of the expression patterns of the neighbouring genes of the operon, the *aadA* gene was integrated in the same transcriptional orientation as *psaI* (Fig. 1A; see ‘Materials and methods’). Plastid transformation was performed using the biolistic method (Svab and Maliga, 1993) and selection for spectinomycin resistance yielded several independent transplastomic lines (subsequently referred to as *Nt*-∆*psaI* lines). Selection for homoplasmy (i.e. the presence of a homogeneous population of transformed plastid genomes) was performed by conducting three additional rounds of regeneration on spectinomycin-containing medium. Subsequently, the transplastomic lines were rooted and transferred to the greenhouse for seed production. All further analyses were performed with the next generation (T1). To verify homoplasmy of the lines and to test for correct insertion of the selectable marker gene *aadA* into *psaI*, RFLP analyses were performed with four independent transplastomic lines (Fig. 1B). To this end, the plastid genome was digested with the restriction enzymes XhoI and HindIII that cut the *accD* gene upstream of the *psaI* operon and the *ycf10* gene downstream of *psaI*, respectively. In the wild type, this generates a restriction fragment of 3371 bp, while in the transplastomic plants, owing to the *aadA* insertion, a longer fragment of 4471 bp was expected. Indeed, only the smaller fragment was observed in the wild type, while the larger signal was obtained in the transplastomic lines. A weak signal of wild-type size was observed in all transformant lines. This was likely due to the presence of non-functional plastid DNA (so-called promiscuous DNA) in the nuclear genome (Ayliffe and Timmis, 1992; Hager *et al.*, 1999; Stegemann *et al.*, 2003). To ultimately confirm the absence of residual copies of the wild-type plastid genome and thus homoplasmy of the transplastomic *psaI* knockout lines, seed germination assays on spectinomycin-containing medium were performed. While both wild-type and *Nt*-∆*psaI* seedlings were uniformly green when germinated on synthetic medium without spectinomycin, germination on medium with 500 µg mL^−1^ spectinomycin resulted in uniformly white wild-type seedlings and uniformly green *Nt*-∆*psaI* seedlings (Fig. 1C). This result is consistent with the uniparentally maternal inheritance of the plastid genome and ultimately verifies that the wild-type allele of *psaI* was successfully replaced by the knockout allele in all copies of the plastid genome.

Because *psaI* is part of a tetracistronic transcription unit, we next wanted to confirm that disrupting the gene with the *aadA* selectable marker did not disturb the expression of the other three genes in the operon. To this end, northern blot analyses with specific probes for *psaI*, *ycf4*, *ycf10*, and *petA* were performed. The *psaI* probe detected a major transcript of ~0.5 kb in the wild type, which corresponds to the fully processed (monocistronic) *psaI* mRNA. In the transplastomic lines, the probe detected a complex pattern of transcript species. This was due to the hybridization of the probe to the disrupted *psaI* sequences (Fig. 1A), which were part of a large number of polycistronic precursor transcripts, incompletely processed transcripts, and, presumably, degradation intermediates (Fig. 1D). Hybridization to probes for the three genes downstream of the operon revealed the presence of a prominent additional large band that was absent from the wild type and corresponded to the unprocessed tetracistronic precursor RNA that now additionally included 1.1 kb of *aadA* sequence (Fig. 1D). Besides this size shift in the precursor, all mature transcripts and processing intermediates seen in the wild type were also detected in the *Nt*-∆*psaI* transplastomic lines, suggesting that operon expression and mRNA maturation patterns are not disturbed by the disruption of *psaI*. Instead, transcripts from the genes downstream of the operon were clearly overrepresented (Fig. 1D), presumably due to read-through transcription from the promoter driving the *aadA* marker gene.

### No major differences in photosynthetic function of young source leaves under different growth light intensities

To gain insight into the function of the I-subunit, we grew wild-type tobacco and two independent transplastomic lines (named *Nt*-∆*psaI*-1 and *Nt*-∆*psaI*-5) under three different light intensities. All other environmental parameters were kept constant. Plants were germinated under low-light conditions (100 µE m^−2^ s^−1^ light intensity). After 4 weeks, they were either kept in low light or transferred into intermediate (350 µE m^−2^ s^−1^) or high-light conditions (1000 µE m^−2^ s^−1^), where they were grown for 14 days prior to all experiments. Newly developed leaves, which had fully acclimated to the light regimes, were analysed. Photosynthesis in tobacco is strongly light-limited under low light intensities. Therefore, any defect in light harvesting in the ∆*psaI* transformants would result in an observable phenotype under these conditions. In high light, transformants with defects in photosynthetic complex stability and repair or in ROS detoxification by the photosynthetic apparatus should be more prone to photodamage than the wild type.

**Fig. 2.**
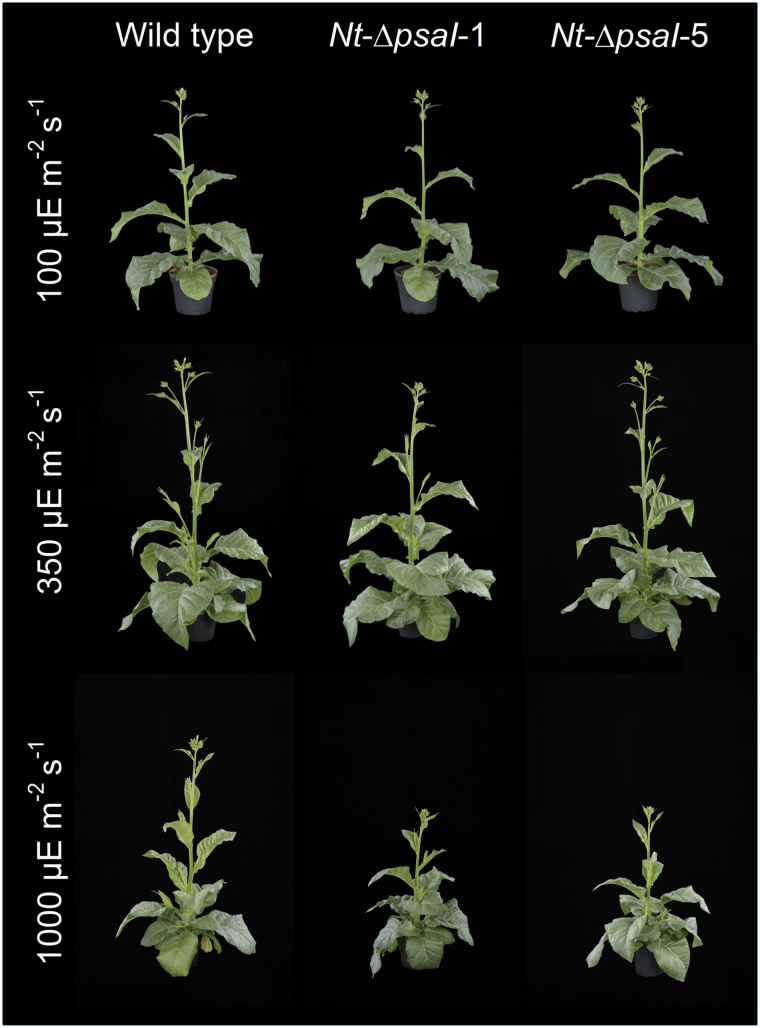
Growth phenotypes of tobacco wild type and two transplastomic ∆*psaI* lines under (**A**) low-light conditions (100 µE m^−2^ s^−1^), (**B**) intermediate-light conditions (350 µE m^−2^ s^−1^), and (**C**) high-light conditions (1000 µE m^−2^ s^−1^). Except for the differences in actinic light intensity, all other environmental parameters such as day length, temperature, and relative humidity were kept constant. While under low and intermediate light intensities, no growth phenotype was observed; under high-light conditions, growth of the two transplastomic tobacco lines was slightly retarded.

**Fig. 3.**
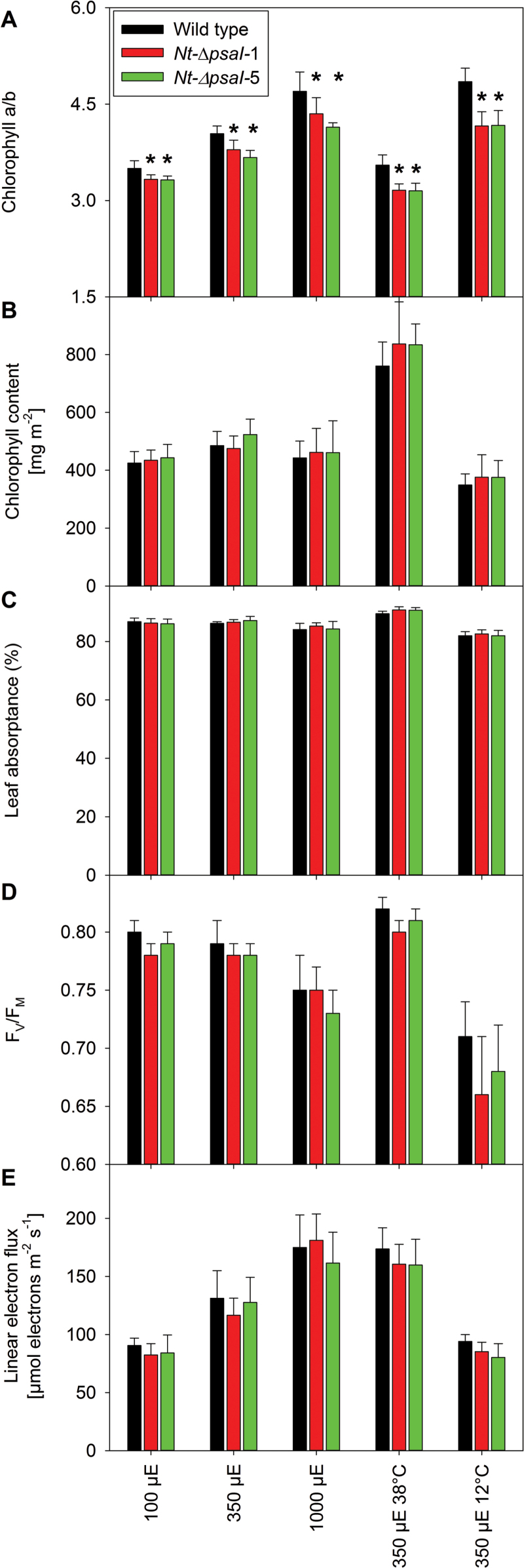
Photosynthetic parameters in wild-type tobacco and two ∆*psaI* mutants. Plants were grown in low-light conditions (100 µE m^−2^ s^−1^), intermediate-light conditions (350 µE m^−2^ s^−1^), high-light conditions (1000 µE m^−2^ s^−1^), and in intermediate-light conditions at an elevated day temperature of 38°C and a decreased temperature of 12°C. All measurements were performed at 22°C. Data are the averages of a minimum of six biological replicates from at least two independent plant cultures, and the standard deviation is shown. The asterisks indicate significant differences between wild type and mutants. (**A**) Chlorophyll a/b ratio. (**B**) Chlorophyll content per leaf area. (**C**) Leaf absorptance. (**D**) F_V_/F_M_, the maximum quantum efficiency of PSII in the dark-adapted state. (**E**) Linear electron flux capacity, which was corrected for leaf absorptance.

**Fig. 4.**
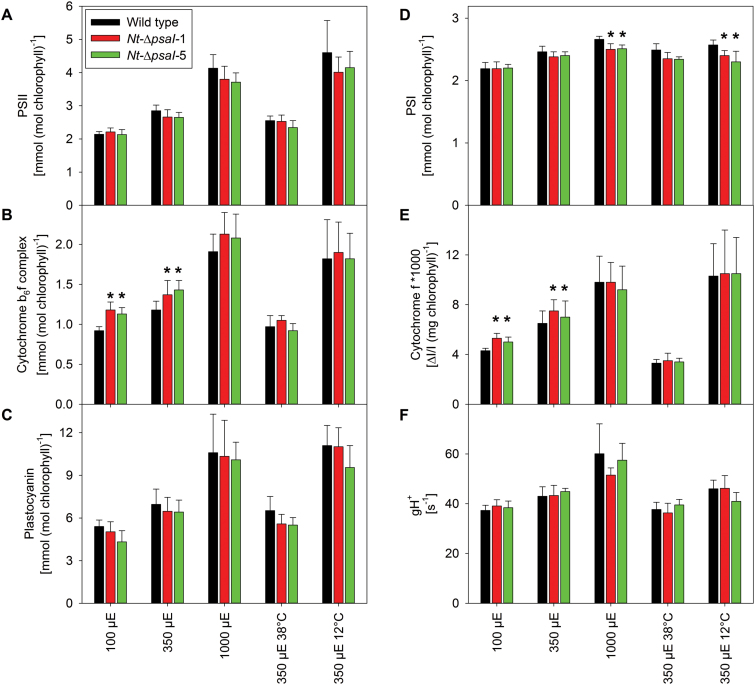
Accumulation levels of the different components of the photosynthetic electron transport chain in wild-type tobacco and two ∆*psaI* mutants. Plants were grown in low-light conditions (100 µE m^−2^ s^−1^), intermediate-light conditions (350 µE m^−2^ s^−1^), high-light conditions (1000 µE m^−2^ s^−1^), and in intermediate-light conditions at an elevated temperature of 38°C and a decreased temperature of 12°C. The components of the electron transport chain were normalized to a chlorophyll basis. Data are the averages of a minimum of six biological replicates from at least two independent plant cultures, and the standard deviation is shown. The asterisks indicate significant differences between wild type and mutants. (**A**) PSII concentration. (**B**) Cytochrome b_6_f complex concentration. (**C**) Plastocyanin concentration. (**D**) PSI concentration. (**E**) Redox-active cytochrome f. (**F**) ATP synthase activity (gH^+^).

**Fig. 5.**
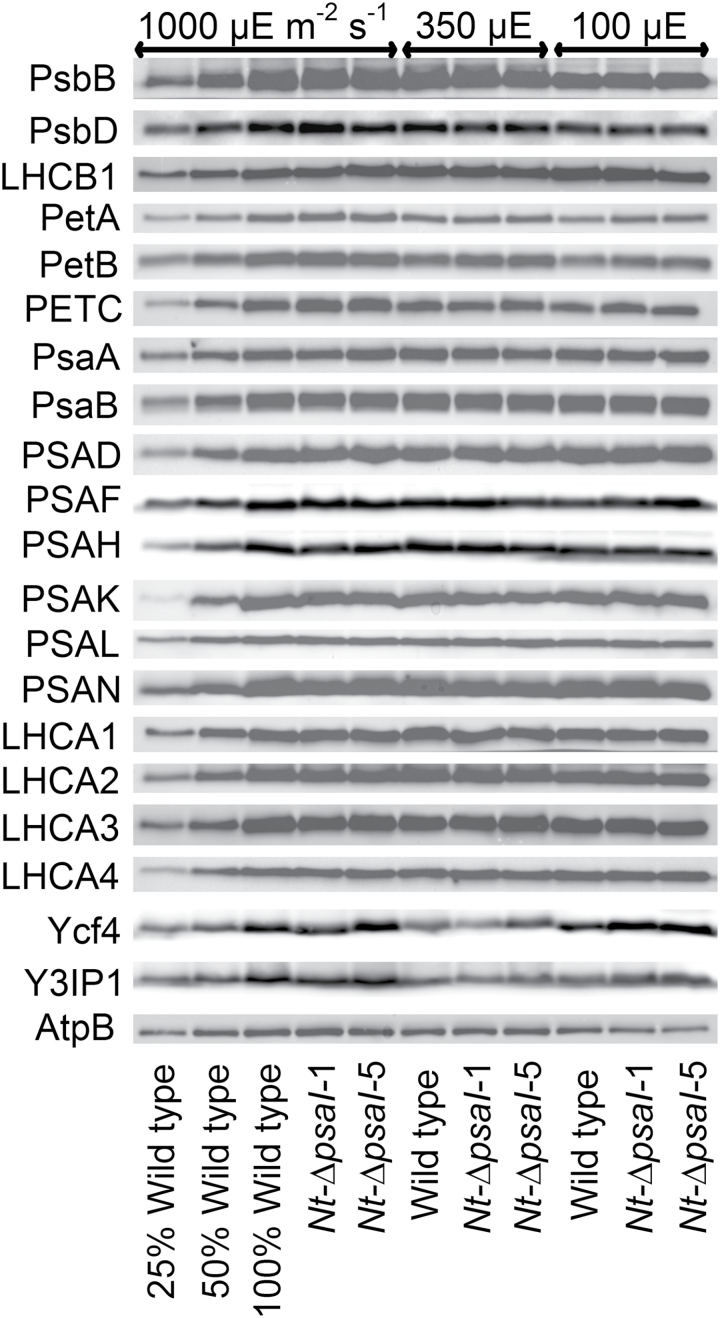
Immunoblot analysis of photosynthetic complex accumulation in wild-type tobacco and the two ∆*psaI* lines grown under low, intermediate, and high-light conditions. Because the accumulation of most tested proteins was highest under high-light conditions, lanes one to three contain samples diluted to 25%, 50%, and a 100% sample of wild-type tobacco grown under high-light conditions, to allow for semi-quantitative determination of changes in protein abundance. Lanes four and five contain the two transplastomic lines grown at 1000 µE m^−2^ s^−1^. Lanes six to eight contain wild-type tobacco and the mutants grown at intermediate light intensities, and lanes nine to eleven contain samples grown at low light intensities. For PSII, the accumulation of the essential subunits PsbB (CP43) and PsbD (D2) and the LHCB1 antenna protein were determined, while for the cytochrome b_6_f complex, the accumulation of the essential redox-active subunits PetA (cytochrome f), PetB (cytochrome b_6_), and PETC (Rieske FeS protein) was tested. AtpB was probed as an essential subunit of the chloroplast ATP. For PSI, in addition to the three essential plastome-encoded subunits PsaA, PsaB, and PsaC, the accumulation of the nuclear-encoded subunits PSAD, PSAH, PSAK, PSAL, and PSAN and of the four LHCI proteins (LHCA1, LHCA2, LHCA3, LHCA4) was determined. Finally, we examined the accumulation of Ycf4, the chloroplast-encoded PSI-biogenesis factor encoded in the same operon as PsaI, and the nuclear-encoded assembly factor Y3IP1.

**Fig. 6.**
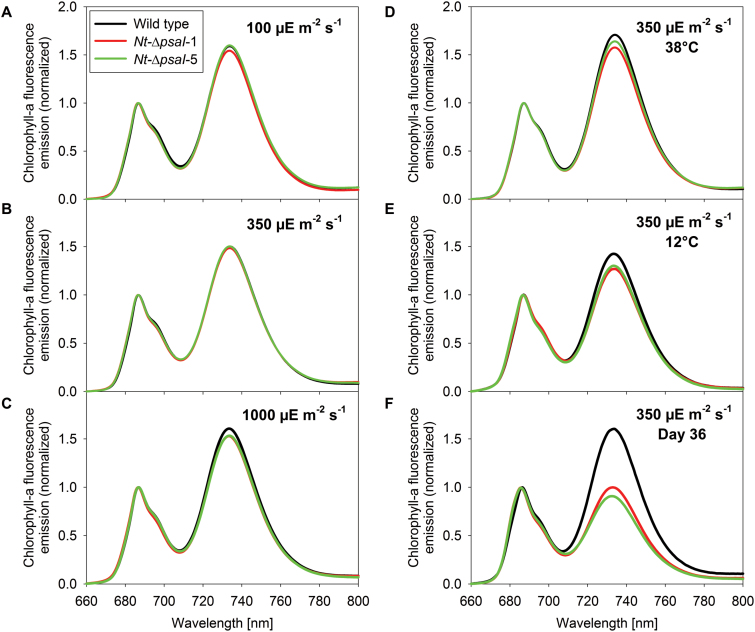
77 K chlorophyll-a fluorescence emission spectra of wild-type tobacco and the two transplastomic *psaI* knockout lines. Plants were grown in (**A**) low-, (**B**) intermediate-, and (**C**) high-light conditions; and at (**D**) intermediate light and 38°C day temperature, (**E**) intermediate light and 12°C day temperature, and (**F**) intermediate light at 22°C for 36 days. No significant differences were observed in the 77 K chlorophyll-a fluorescence emission spectra under the different light regimes and at 38°C growth temperature, confirming that the coupling of the LHC to the reaction centres and the relative antenna cross sections of the photosystems are unaffected by the deletion of the I-subunit. In plants grown at 12°C day temperature and especially in senescing leaves, the loss of PSI is reflected by the decrease of the PSI-LHCI emission signal at 733 nm wavelength. Data are the averages of a minimum of six biological replicates from at least two independent plant cultures.

Under all conditions, plants were grown until the wild type started to flower, and then photographed. Different from the growth retardation in low light reported by Plöchinger *et al.* (2016), our transplastomic lines did not show a visible phenotype when grown at 100 or 350 µE m^−2^ s^−1^ actinic light intensity (Fig. 2, top and middle row), but when raised under 1000 µE m^−2^ s^−1^, their growth was slightly retarded and the onset of flowering was delayed (Fig. 2, lower row). To determine if the appearance of the high-light growth phenotype could be ascribed to a photosynthetic defect that is especially pronounced at 1000 µE m^−2^ s^−1^ actinic light intensity, detailed biochemical and biophysical analyses were performed with the wild type and the two transplastomic lines grown under the different light environments. We first determined several photosynthetic parameters on intact leaves (Fig. 3), followed by thylakoid isolation and quantification of the components of the electron transport chain via difference absorbance spectroscopy (Fig. 4) and immunoblotting (Fig. 5). Photosynthetic complexes were determined on a chlorophyll basis, which is a good proxy for changes in complex contents on a thylakoid membrane level (Schöttler and Tóth, 2014). Also, 77 K chlorophyll-a fluorescence emission spectra were recorded on isolated thylakoids to assess the relative antenna cross sections of the photosystems (Fig. 6A–C).

We found clear differences in many photosynthetic parameters between plants grown under the different light regimes, but observed only few significant differences between the wild type and the mutants. Increases in light intensity led to increases in the chlorophyll a/b ratio (Fig. 3A), and approximately 2-fold increases in linear electron transport capacity (Fig. 3E) and in PSII (Fig. 4A), b_6_f (Fig. 4B), and plastocyanin (Fig. 4C). These findings are in line with known light acclimation responses described for tobacco and many other plant species (Schöttler and Tóth, 2014). The conductivity of the thylakoid membrane for protons (gH^+^), a measure for chloroplast ATP synthase activity, also increased with light intensity (Fig. 4F). The chlorophyll content per leaf area (Fig. 3B), leaf absorptance (Fig. 3C), and the content of PSI (Fig. 4D) did not show pronounced light-intensity-dependent changes. The maximum quantum efficiency of PSII, F_V_/F_M_, decreased only slightly at the highest actinic light intensity (Fig. 3D), indicating that even when grown at 1000 µE m^−2^ s^−1^, tobacco does not suffer from strong PSII photoinhibition.

When comparing the wild type to the two ∆*psaI* lines, the only significant difference observed at all light intensities was a lower chlorophyll a/b ratio in the mutants (Fig. 3A). This indicates a change in the ratio of photosynthetic reaction centres (which only bind chlorophyll a) to their antenna proteins (which bind both chlorophyll a and chlorophyll b). Accordingly, in high light, a significant decrease in PSI content was observed in both transplastomic lines, suggesting that the I-subunit plays a role in PSI stability under light-stress conditions (Fig. 4D). Surprisingly, the content of the b_6_f was significantly higher in the transformants when grown in low and intermediate light intensities (Fig. 4B). To determine if the increased amount of b_6_f was fully active in electron transport, the difference between the transmission signals of fully oxidized and fully reduced cytochrome f (at the end of a saturating light pulse and after a short interval of darkness, respectively) was measured on intact leaves and normalized to the chlorophyll content. Indeed, significantly increased amplitudes of the cytochrome f difference transmission signal were obtained for the mutants grown in low and intermediate light (Fig. 4E).

Photosynthetic complex accumulation was also analysed by immunoblotting using isolated thylakoids (Fig. 5). In line with our spectroscopic results, the contents of the two essential PSII subunits PsbB (CP43) and PsbD (D2 protein) were highest in plants grown under high-light conditions, and declined with decreasing actinic light intensity. The LHCII subunit LHCB1 behaved in the opposite way, showing increased contents in low light. However, we did not observe any differences in PSII contents between the wild type and the transplastomic lines. As expected, protein levels of the b_6_f subunits PetA (cytochrome f), PetB (cytochrome b_6_), and PETC (the Rieske protein) increased with the actinic light intensity. For PSI, we probed the essential plastid-encoded reaction centre subunits PsaA and PsaB, as well as the nuclear-encoded subunits PSAD, PSAF, PSAH, PSAK, PSAL, and PSAN. We did not observe differences in PSI subunit accumulation between the wild type and the transplastomic lines under any light condition. Presumably, the small reduction in redox-active PSI determined spectroscopically for plants grown in high-light conditions (Fig. 4D) was below the resolution of the immunoblots. We found no differences dependent on the growth light intensity or the genotype for the four nuclear-encoded LHCI proteins (LHCA1–LHCA4). Because mRNA accumulation of *ycf4* was increased in the ∆*psaI* transformants (Fig. 1D), we checked if it had increased protein accumulation as well. This was the case only in low-light-acclimated mutants. We also observed an unusual light-intensity effect on Ycf4 accumulation: Ycf4 abundance was highest in high-light conditions, and showed an approximate 75% reduction when grown at 350 µE m^−2^ s^−1^. However, at the lowest light intensity of 100 µE m^−2^ s^−1^, Ycf4 content was almost as high as under high-light conditions. For Y3IP1 (Albus *et al.*, 2010), another assembly factor for PSI, we did not observe any differences between the wild type and the transplastomic lines. Its abundance was highest at 1000 µE m^−2^ s^−1^, and much lower at the two other light intensities. Finally, we did not see any differences in chloroplast ATP synthase between the wild type and the mutants, but, similar to ATP synthase activity (Fig. 4F), AtpB concentration increased in high-light-acclimated plants.

### *Antenna organization and light harvesting are unaltered in the* ∆psaI *transformants*

To assess possible differences in antenna organization of the photosystems, 77K chlorophyll-a fluorescence emission spectra were measured. They did not reveal clear differences between the wild type and the transplastomic lines (Fig. 6A–C). The emission maxima of both PSII (at 686-nm wavelength) and PSI (at 733-nm wavelength) were unaltered, and the area below the two emission signals relative to each other did not change significantly. Most importantly, all LHCI proteins were efficiently coupled to the PSI reaction centre (free, uncoupled LHCI proteins would give rise to additional fluorescence emission bands between 705 and 725 nm, which we did not observe).

**Fig. 7.**
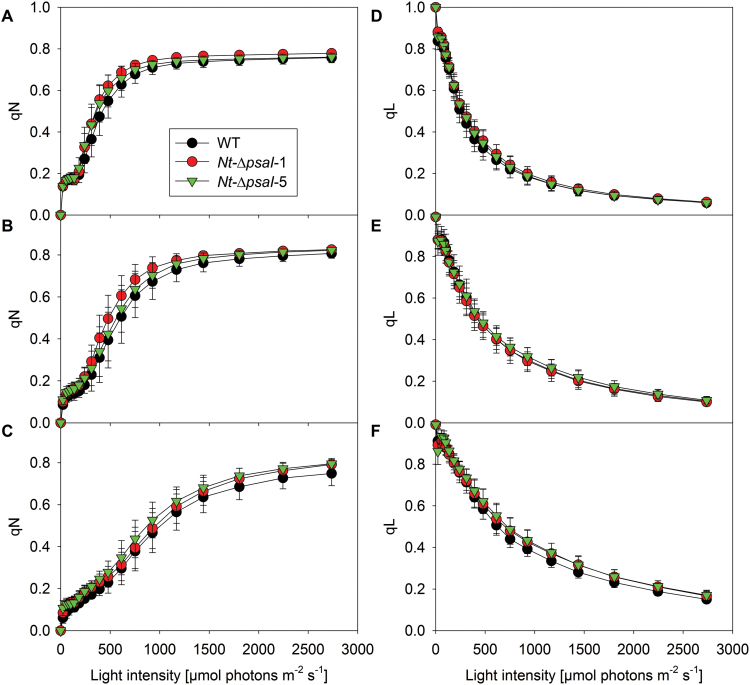
Light-response curves of chlorophyll-a fluorescence parameters. (**A–C**) qN is a measure of non-photochemical quenching of excess excitation energy. (**D–F**) qL is a measure of the redox state of the PSII acceptor side. No significant differences were observed under low (A, D), intermediate (B, E), or high (C, F) light intensities between the wild type (WT) and the transplastomic mutants. Data are the averages of a minimum of six biological replicates from at least two independent plant cultures, and the standard deviation is shown.

An unaltered antenna structure was also supported by the light-response curves of chlorophyll-a fluorescence parameters (Fig. 7). qN is a measure of the induction of photoprotective mechanisms harmlessly dissipating excess excitation energy as heat. qL is a measure of the redox state of the PSII acceptor side (Kramer *et al.*, 2004). Light-response curves of both parameters revealed no significant differences between the wild type and the mutants in the different growth light regimes. Increased growth light intensities resulted in a shift in the induction of qN and in the reduction of the PSII acceptor side to higher light intensities, reflecting the increased photosynthetic capacity of these plants (Fig. 3E).

**Fig. 8.**
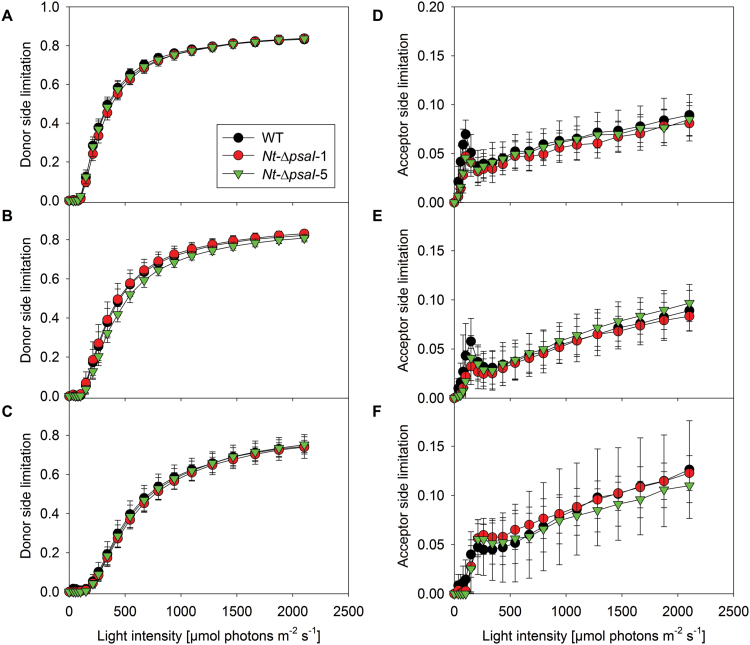
Light-response curves of the (**A–C**) donor-side limitation and the (**D–F**) acceptor-side limitation of PSI. No significant differences were observed for plants grown under low (A, D), intermediate (B, E), or high (C, F) light intensities between the wild type (WT) and the two transplastomic lines. Data are the averages of a minimum of six biological replicates from at least two independent plant cultures, and the standard deviation is shown.

The light-response curves of the donor-side limitation of PSI (Fig. 8A–C) also reflected this increase in electron transport capacity with increasing growth light intensity. PSI becomes donor-side limited whenever the electron transfer capacity from PSII to PSI is lower than the excitation rate of PSI, so that P_700_^+^ starts to accumulate. Again, no differences were observed between the wild type and the two knockout lines (Fig. 3E). We additionally analysed the acceptor-side limitation of PSI (Fig. 8D–F), which arises either from an impaired forward electron transfer towards ferredoxin, or from an altered capacity of the Calvin–Benson cycle to consume the NADPH produced by the light reactions. The acceptor-side limitation tended to increase with the actinic light intensity, but never exceeded more than 10% of the total PSI population. We did not observe significant differences between the wild type and the transplastomic lines from any of the three light regimes, suggesting that forward electron transfer from PSI to ferredoxin and NADP^+^ works normally in the *psaI* knockout plants.

A sensitive measure for even minor impairments in light harvesting is the quantum efficiency of CO_2_ fixation. We, therefore, performed gas exchange measurements under CO_2_-saturated conditions (2000 ppm CO_2_) to repress photorespiration and the Mehler reaction. In addition to the quantum efficiency of CO_2_ fixation, we also determined leaf respiration in darkness and the maximum light-saturated assimilation capacity (Table 1; Supplementary Fig. S1A, available at *JXB* online). These analyses were restricted to plants grown at 350 µE m^−2^ s^−1^. Respiration in darkness and the assimilation capacity were not significantly different between the wild type and the ∆*psaI* lines, and all needed approximately 12 quanta per assimilated molecule of CO_2_ (Table 1; Supplementary Fig. S1B). This value is in the expected range for higher plants measured under CO_2_-saturated conditions (Rott *et al.*, 2011; Albus *et al.*, 2012).

**Table 1.** Gas exchange parameters, state transitions, and redox equilibration between plastocyanin and P _700
_ Leaf gas exchange was measured on the youngest fully expanded leaf of plants grown at 350 µE m^−2^ s^−1^ light intensity. All measurements were performed at 22°C and in saturating CO_2_ (2000 ppm) to repress photorespiration and determine the maximum quantum efficiency of photosynthetic CO_2_ assimilation. Leaf respiration was measured after a minimum of 30 min of dark adaptation. Then, the light intensity was stepwise increased until light saturation of photosynthesis was reached, to determine the maximum capacity of leaf assimilation. Assimilation capacity was corrected for dark respiration. The maximum quantum efficiency of CO_2_ assimilation was calculated in the linear range of the light response curve, between 0 and 100 µE m^−2^ s^−1^ actinic light intensity. The capacity to perform state transitions is expressed as the F_R_ parameter (Lunde *et al.*, 2000). Redox equilibration between plastocyanin and P_700_ during a short dark interval interrupting steady-state photosynthesis in saturating light is described by the apparent redox equilibrium constant, K_app_ (according to Kirchhoff *et al.*, 2004).

Parameter	Wild type	*Nt*-∆*psaI-1*	*Nt*-∆*psaI-5*
Respiration [µmol CO_2_ m^−2^ s^−1^]	−1.5 ± 0.3	−1.6 ± 0.3	−1.6 ± 0.5
Assimilation capacity [µmol CO_2_ m^−2^ s^−1^]	31.4 ± 3.1	31.6 ± 4.1	32.0 ± 4.5
Quanta / CO_2_	12.3 ± 0.8	11.9 ± 0.8	12.2 ± 1.0
F_R_	0.72 ± 0.06	0.77 ± 0.08	0.81 ± 0.05
K_app_	14.6 ± 2.3	16.0 ± 2.1	16.3 ± 1.9

Finally, to determine a possible role of the I-subunit in docking of the LHCII trimer under state-2 conditions, we assayed the capacity of the wild type and the ∆*psaI* lines to perform state transitions. Leaves were illuminated for 15 min with weak light, preferentially exciting PSII-LHCII (enriched in blue light, to induce a switch to state 2), followed by a 15-min illumination with weak light, preferentially exciting PSI (enriched in far-red light, to induce a switch back to state 1), and changes in steady-state chlorophyll-a fluorescence were recorded. Example chlorophyll-a fluorescence kinetics are shown in Supplementary Fig. S1C. Then, the F_R_ parameter was calculated according to Lunde *et al.* (2000). No differences in the amplitudes of state transitions could be identified (Table 1).

### Redox equilibration between plastocyanin and P_700_

To determine if the loss of the I-subunit interferes with the binding and oxidation of plastocyanin, we determined the redox equilibration between P_700_ and plastocyanin in intact plants grown in the intermediate-light regime. Because light-intensity-dependent changes in the thylakoid lumen diameter affect plastocyanin diffusion (Kirchhoff *et al.*, 2011), leaves were pre-illuminated with saturating light. This also ensured that the Calvin–Benson cycle was fully activated, so that an acceptor-side limitation of PSI was avoided. A short saturating light pulse (5000 µE m^−2^ s^−1^ for 250 ms) was then applied to achieve full oxidation of both plastocyanin and P_700_. At the end of that light pulse, the actinic illumination was switched off, and the reduction kinetics of plastocyanin and P_700_ were measured (Supplementary Fig. S2A, available at *JXB* online). In accordance with the higher redox potential of P_700_, a net reduction of plastocyanin only occurred after the vast majority of P_700_ was already reduced. To quantitatively describe the interaction between plastocyanin and P_700_, the normalized reduction kinetics were plotted against each other (Supplementary Fig. S2B), and an apparent redox equilibration constant (K_app_) was calculated (Table 1; Kirchhoff *et al.*, 2004; Schöttler *et al.*, 2007a). No significant differences between the wild type and the transplastomic lines could be determined, strongly indicating that loss of the I-subunit does not affect plastocyanin binding and oxidation.

### Light-induced pmf formation and cyclic electron transport

**Fig. 9.**
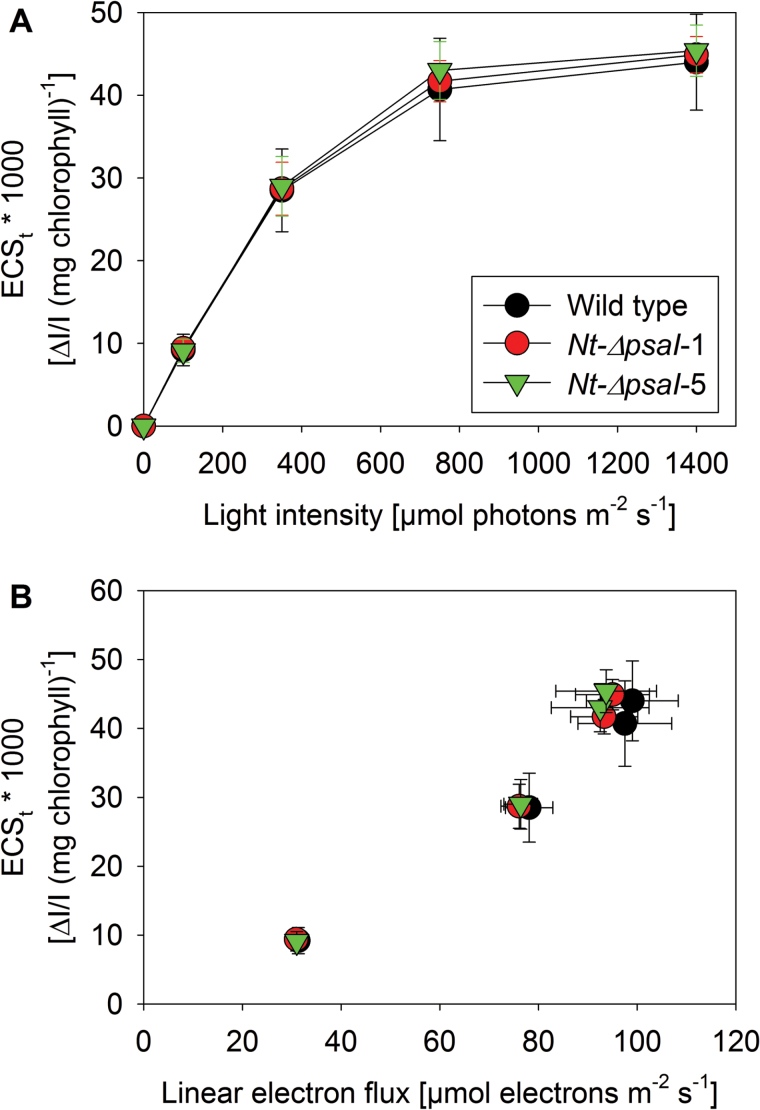
Light-response curve of the light-induced pmf across the thylakoid membrane (ECS_t_) (**A**), and dependence of the light-induced pmf on linear electron transport (**B**). Data are the averages of eight biological replicates from two independent plant cultures, and the standard deviation is shown.

To assess possible differences in the light-induced pmf formation across the thylakoid membrane and in cyclic electron transport around PSI in the ∆*psaI* mutants, we measured light-response curves of the total amplitude of the electrochromic shift signal (ECS_t_; Fig. 9A) in plants grown in intermediate light. These measurements did not reveal any significant differences between the wild type and the mutants. We then calculated the dependence of the light-induced pmf at each of the actinic light intensities on the linear electron flux rate, determined simultaneous to the ECS_t_ measurements and corrected for leaf absorptance (Fig. 9B). To this end, we plotted ECS_t_ as a function of linear electron flux. Again, no significant differences were observed. Together with the fact that ATP synthase activity was indistinguishable between the wild type and the mutants (Fig. 4F), these data clearly show that cyclic electron flux was unaltered: a higher cyclic electron flux would have resulted in an increased ECS_t_, relative to linear electron flux, while a decreased cyclic flux would have resulted in a lower ECS_t_. We conclude that at all light intensities, either no or a constant proportion of cyclic electron flux, relative to linear electron flux, occurs in the mutants and the wild type.

### Plant growth at 12°C and 38°C

Under high-light conditions, we saw a slight growth retardation (Fig. 2), a tendency towards a decreased maximum quantum efficiency of PSII (Fig. 3D), and a significant decrease in PSI content (Fig. 4D) in the ∆*psaI* mutants. Because high light results in both increased leaf temperature and a general increase in photosynthetic ROS production, we decided to dissect a possible heat effect on the photosynthetic performance of the mutants from an increased ROS production or an increased susceptibility of PSI to oxidative damage. To this end, plants were grown at the intermediate light intensity of 350 µE m^−2^ s^−1^, but at increased (38°C) and decreased (12°C) temperatures. To obtain comparable data on the activity of electron transport, we performed all activity measurements at 22°C.

In most higher plants, photosynthesis suffers massively within a few minutes to hours after transfer to temperatures of 10–15°C above ambient temperature. This has been ascribed to multiple different mechanisms, including direct damage to the photosynthetic apparatus, such as uncoupling of the thylakoid membrane, and specific damage to PSII (reviewed by Allakhverdiev *et al.*, 2008; Sharkey and Zhang, 2010; Schöttler and Tóth, 2014). Because we had previously observed that wild-type tobacco tolerates long-term growth even at temperatures up to 38°C (Wolfram Thiele and Mark Aurel Schöttler, unpublished data), we transferred 4-week-old plants to 38°C day temperatures and 34°C night temperatures. The first leaves that newly developed under these conditions (and thus were fully acclimated to the elevated temperatures) were analysed 2 weeks later.

No significant differences were observed between the wild type and the knockout lines, except for the decreased chlorophyll a/b ratio (Fig. 3A). Chlorophyll content per leaf area (Fig. 3B), leaf absorptance (Fig. 3C), F_V_/F_M_ (Fig. 3D), linear electron flux capacity (Fig. 3E), photosynthetic complex accumulation and chloroplast ATP synthase activity (Fig. 4A–F) were indistinguishable. We also observed no significant differences for the antenna distribution between the photosystems (Fig. 6D). However, the chlorophyll content per leaf area increased to more than 160% in all plants, relative to plants grown at 22°C (Fig. 3B). This was due to a strong increase in leaf thickness. The increased chlorophyll content suggests a proportional increase of all complexes per leaf area, explaining the higher capacity of linear electron transport in plants grown at 38°C versus plants grown at 22°C (Fig. 3E).

When plants are exposed to low temperatures, photosynthetic ROS production is strongly increased owing to an imbalance between photosynthetic electron transport, which has low temperature dependence, and primary metabolism, which is strongly slowed down. Metabolic ATP and NADPH consumption decreases, electrons accumulate in the electron transport chain, and ROS production especially on the PSI acceptor side is increased, leading to PSI photoinhibition (Scheller and Haldrup, 2005). Therefore, we decided to challenge the ∆*psaI* mutants with a combination of a chilling stress (12°C during the day, 8°C during the night) and 350 µE m^−2^ s^−1^ light intensity.

First, we followed the fate of leaves that had already developed prior to the transfer from the low-light chamber (100 µE m^−2^ s^−1^, 22°C day temperature) to the combination of intermediate-light and chilling stress (Supplementary Fig. S3, available at *JXB* online). Their length only increased marginally over 14 days of chilling stress (Supplementary Fig. S3A). The chlorophyll content per leaf area (Supplementary Fig. S3C), linear electron flux capacity (Supplementary Fig. S3E), and, especially, F_V_/F_M_ (Supplementary Fig. S3D) decreased in both wild type and mutants during the first days of chilling stress. However, after 7–14 days of chilling stress, differences started to occur, in that both linear electron flux capacity and F_V_/F_M_ recovered in the wild type whereas no such recovery was observed in the mutants. We could not perform a full analysis of photosynthetic complex accumulation with these leaves because a massive accumulation of starch precluded the isolation of thylakoids.

Instead, we analysed the newly developed leaves in detail. Because of strongly decreased plant growth at 12°C, these leaves were only measured after 3 weeks of chilling stress. Both in the wild type and in the mutants, F_V_/F_M_ was clearly reduced (Fig. 3D) relative to plants grown at 22°C and 38°C, confirming that chilling also increased oxidative damage to the photosynthetic apparatus in the newly developed leaves. We again found a significant decrease in the chlorophyll a/b ratio (Fig. 3A). Additionally, PSI concentration was significantly decreased (Fig. 4D). In accordance with this observation, the PSI emission signal at a wavelength of 733 nm showed a small but significant decrease (Fig. 6E).

### Leaf ontogenesis

**Fig. 10.**
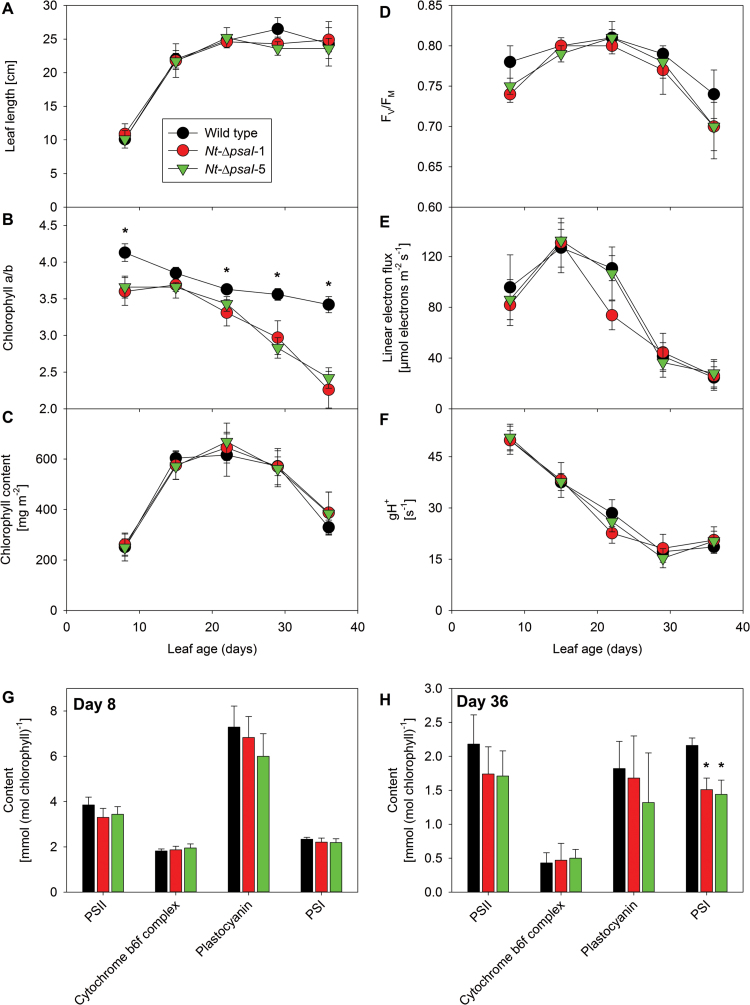
Changes in photosynthetic parameters during leaf ontogenesis. (**A**) Leaf length. (**B**) Chlorophyll a/b ratio. (**C**) Chlorophyll concentration. (**D**) Maximum quantum efficiency of PSII in the dark-adapted state (F_V_/F_M_). (**E**) Linear electron flux (corrected for leaf absorptance). (**F**) ATP synthase activity (gH^+^). (**G**) Photosynthetic complex accumulation per chlorophyll in young leaves (8 days old). (**H**) Photosynthetic complex accumulation per chlorophyll in senescing leaves (36 days old). Data are the averages of a minimum of six biological replicates from three independent plant cultures, and the standard deviation is shown. The asterisks indicate significant differences between wild type and mutants.

We next tested for differences between the wild type and the ∆*psaI* mutants during leaf development. We followed the development of the true leaf number five, the first leaf that developed after the transfer of 4-week-old plants to the intermediate light intensity of 350 µE m^−2^ s^−1^. Photosynthetic parameters of the leaf were first determined on day eight of its development, when it was large enough for us to perform all spectroscopic measurements. We then re-measured the same leaf after 15, 22, 29, and 36 days, when the leaf became visibly senescent. One week later, the leaves of all plants had lost turgor and did not show any measurable photosynthetic activity. Therefore, this time point was not included in the detailed characterization of ontogenetic changes. As a proxy of leaf development, we determined leaf length (Fig. 10A), which increased until day 22, when the leaf was fully expanded. No differences in leaf length were observed between the wild type and the transplastomic lines. In line with the full leaf expansion being reached at day 22, the chlorophyll content per leaf area peaked on this day, and then started to decline (Fig. 10C). F_V_/F_M_ showed only minor changes with leaf age, but tended to decrease more strongly in the mutants at day 36 (Fig. 10D). Linear electron flux capacity peaked on day 15 of leaf development, and then decreased to less than 20% of the maximum rate on day 36, but again, no clear differences occurred between the wild type and the mutants (Fig. 10E). The chloroplast ATP synthase activity was highest on day 8 of leaf development and decreased to 35% of the maximum rate with increasing leaf age (Fig. 10F). In the wild type, the chlorophyll a/b ratio was highest on day 8, and then decreased continuously, indicating that the contents of antenna proteins strongly increased relative to the reaction centres (Fig. 10B). In both transplastomic knockout lines, the a/b ratio was significantly lower than in the wild type on all days except for day 15 of leaf development. The differences were most pronounced on day eight, when the leaves were still establishing their photosynthetic apparatus, and on day 36, when leaf senescence had begun and the chlorophyll a/b ratio of the wild type was 3.2 compared to 2.3 in the mutant lines.

Therefore, we decided to investigate the composition of the photosynthetic apparatus at these two time points in detail. The contents of PSII, the b_6_f, and plastocyanin were between 2- and 4-fold higher in young (Fig. 10G) than in old leaves (Fig. 10H). For the PSI content, no leaf age-dependent changes were observed in the wild type. However, PSI contents tended to be lower in the ∆*psaI* mutants. This difference was significant at day 36, when the difference in the chlorophyll a/b ratios was most pronounced. PSI accumulation was reduced from 2.2 mmol per mol chlorophyll in the wild type to less than 1.5 mmol per mol chlorophyll in the mutants, clearly demonstrating a reduced stability and/or accelerated degradation of redox-active PSI during leaf senescence. This accelerated loss of PSI also resulted in a drastically diminished 77 K chlorophyll-a fluorescence emission signal from PSI-LHCI, relative to that from PSII-LHCII (Fig. 6F).

### In vitro *stability of PSI*

A specific role for the I-subunit in stabilizing the binding of PSAL and PSAH has previously been suggested based on the known PSI structure (Jensen *et al.*, 2007; Amunts *et al.*, 2010). In our immunoblot analyses (Fig. 5), we did not see any evidence for a loss of these two subunits, but could not fully exclude that they dissociated from PSI and accumulated in their free, unbound form in the thylakoid membrane. To test for such an impaired binding of the H- and L-subunit to PSI, we solubilized isolated thylakoids from plants grown under intermediate light in DDM and separated the photosynthetic complexes by discontinuous SDG. Thirty fractions were harvested from the bottom to the top of each gradient, with fraction 1 corresponding to the highest and fraction 30 corresponding to the lowest sucrose density. Proteins were extracted from each fraction and separated by denaturing SDS-PAGE (Supplementary Fig. S4, available at *JXB* online). The distribution of the different complexes in the SDG was determined by immunoblotting against PsbD as a diagnostic subunit for PSII, against LHCB4, and against the PSI subunits PsaA, PSAH and PSAL. The four fractions of highest density did not contain any protein complexes and were not analysed by immunoblotting. We did not observe any differences between wild-type plants and mutants. Most importantly, there was no indication of the presence of free H- or L-subunits, as both subunits strictly co-migrated with PsaA in the gradients.

### Oxidative damage to thylakoid proteins

The tendency towards decreased F_V_/F_M_ values under high-light and chilling stress (Fig. 3D; Supplementary Fig. S3D) and the parallel reduction in PSI content (Fig. 4D) suggest that the mutants might be more sensitive to oxidative damage of the photosynthetic apparatus than the wild type. To test if this is indeed the case, we determined thylakoid protein oxidation with the Oxyblot protein oxidation detection kit (Supplementary Fig. S5, available at *JXB* online). Equal amounts of chlorophyll were loaded for each sample. Comparing protein oxidation signals from plants from low, intermediate, and high-light conditions, we observed a clear increase in protein oxidation with increasing growth light intensity. More important was the clear tendency towards stronger protein oxidation signals in the mutants, relative to the wild type.

## Discussion

The small plastome-encoded PsaI protein is the only subunit of PSI whose precise physiological function has not been elucidated in higher plants to date. Based on the known crystal structure of PSI in pea (Amunts *et al.*, 2010; Mazor *et al.*, 2015; Qin *et al.*, 2015), PsaI has been implicated in the binding of PSAH and PSAL, which constitute the LHCII docking site under state-2 conditions (Jensen *et al.*, 2007; Amunts *et al.*, 2010). A recent study of PSI from a tobacco ∆*psaI* mutant suggests that the I-subunit is indeed required for stable binding of PSAH and PSAL, as evidenced by these subunits migrating as free proteins separate from PSI in native gels (Plöchinger *et al.*, 2016). However, *in vivo*, the authors could not detect any defects in state transitions in their ∆*psaI* mutant. The mutant showed a minor delay in growth, but was indistinguishable from wild-type tobacco in all photosynthetic parameters reported. Because in that study, plants were grown under light-limited conditions of 125 µE m^−2^ s^−1^, a more pronounced role of the I-subunit under different growth conditions and environmental stresses cannot be excluded. Here, we demonstrate that the I-subunit indeed plays a role in the stability of the entire redox-active PSI complex in response to high-light and chilling stress, and this effect is even more pronounced during leaf senescence.

### Increased accumulation of the b_6_f is not a specific response to the loss of the I-subunit

In the ∆*psaI* transplastomic mutants, mRNA accumulation from the three genes downstream of the operon was increased (Fig. 1D). Therefore, any phenotype observed in the ∆*psaI* transformants could, in theory, also result from overexpression of Ycf4, Ycf10, and the PetA subunit of the b_6_f. In particular, overexpression of Ycf4 could have an effect on PSI, because it functions as a non-essential auxiliary protein in late stages of PSI assembly (Krech *et al.*, 2012). We determined Ycf4 protein abundance via immunoblotting (Fig. 5). Ycf4 protein accumulation was slightly increased in the ∆*psaI* transformants when plants were grown in low light, but no differences in protein accumulation were observed between the ∆*psaI* lines and wild-type tobacco in intermediate and high light. Thus, the relatively strong increase in *ycf4* transcript abundance does not translate to a similar increase in Ycf4 protein accumulation, a finding that is consistent with plastid gene expression being predominantly controlled at the post-transcriptional level (Eberhard *et al.*, 2002; Kahlau and Bock, 2008). Ycf10, a non-essential protein located in the inner envelope membrane, has been implicated in inorganic carbon uptake into the chloroplast of *Chlamydomonas reinhardtii* (Rolland *et al.*, 1997). From an ongoing study of ∆*ycf10* transformants in tobacco, it has become clear that it does not play any role in photosynthesis under ambient CO_2_ concentrations, as used for the growth of the ∆*psaI* mutants. However, at low growth light intensities, ∆*ycf10* plants show an increased accumulation of the b_6_f (Claudia Flügel and Mark Aurel Schöttler, unpublished data), similar to the ∆*psaI* lines (Figs 4 and 5). Owing to insertion of the *aadA* gene into *ycf10*, the ∆*ycf10* plants show the same transcriptional upregulation of *petA* as our ∆*psaI* plants (Fig. 1A, D; our unpublished results). Therefore, we ascribe the increased accumulation of the b_6_f to the increased *petA* transcript abundance. Under low-light intensities, b_6_f biogenesis in the wild type might be limited by *petA* mRNA accumulation. Interestingly, despite a similar increase in *petA* transcript abundance in their ∆*psaI* mutant, Plöchinger *et al.* (2016) did not detect an increase in b_6_f content. It is possible that immunoblot analyses are not sufficiently sensitive to detect the relatively small increase in b_6_f, which we have confirmed here by two independent spectroscopic approaches (Fig. 4B, E).

Normally, the b_6_f catalyses the rate-limiting step of linear electron transport (reviewed by Anderson, 1992; Schöttler and Tóth, 2014). Therefore, the increased b_6_f content in our mutants should have resulted in an increased capacity for photosynthetic electron transport in the ∆*psaI* mutants. This was not the case (Fig. 3E), likely because plastocyanin content (Schöttler *et al.*, 2004) and ATP synthase activity (Rott *et al.*, 2011) also contribute to photosynthetic flux control in tobacco. Out of these three components, only the b_6_f was increased here, while plastocyanin content (Fig. 4C) and ATP synthase activity (Fig. 4F) remained unaltered. One could speculate that the additional b_6_f might function in cyclic instead of linear electron flux. However, cyclic electron flux was also not increased in the ∆*psaI* mutants, as demonstrated by an unaltered light-response curve of the light-induced pmf (Fig. 9A), and the identical dependence of pmf formation on linear electron flux (Fig. 9B). These data strongly suggest that the function of PsaI is unrelated to cyclic electron transport.

### The I-subunit is required for PSI stability

Based on crosslinking studies and crystallographic data, the I-subunit has been suggested to specifically stabilize the binding of the nuclear-encoded H- and L-subunits, which are required for LHCII binding during state transitions (Jensen *et al.*, 2007; Amunts *et al.*, 2010). Plöchinger and co-workers (2016) reported that in their tobacco ∆*psaI* mutant, a significant fraction of both PSAH and PSAL detached from PSI in native gels. They concluded that PsaI specifically stabilizes the LHCII docking site of PSI. However, it seems questionable if binding of PSAH and PSAL to PSI is also compromised *in vivo*. Normally, free subunits are prone to rapid proteolytic degradation, as shown by the strong reduction in PSAL accumulation in PSAH-deficient *A. thaliana* mutants (Lunde *et al.*, 2000; Varotto *et al.*, 2002). However, neither Plöchinger et al. (2016) nor we observed any indication of a selective loss of either the H- or L-subunit in immunoblot analyses (Fig. 5). Furthermore, loss of either the H- or the L-subunit strongly compromises state transitions in the respective *Arabidopsis* mutants, and state transitions are not perturbed in the ∆*psaI* mutants (Plöchinger *et al.*, 2016; Table 1; Supplementary Fig. S2C). When we analysed the attachment of PSAH and PSAL to PSI by SDGs (Supplementary Fig. S4), which likely is a milder treatment of complexes than native electrophoresis, both subunits perfectly co-migrated with the reaction centre protein PsaA, thus ultimately excluding the presence of free, uncoupled protein *in vivo*. Along this line, Plöchinger *et al.* (2016) also concede it likely that the detachment of PSAH and PSAL represents a solubilization effect.

Instead, the main function of the I-subunit in higher plants seems to be related to the general stability of PSI. Both high-light and chilling conditions (Fig. 4D) resulted in significant decreases in PSI concentration, but the effect was most pronounced during senescence (Fig. 10H). This does not necessarily indicate that the I-subunit plays a more important role in PSI stability during senescence. PSI is an exceptionally stable complex, and its biogenesis is largely restricted to young leaves: the expression of two auxiliary proteins involved in PSI biogenesis, Ycf4 and Y3IP1, is strongly reduced in mature and old leaves, suggesting that senescing tobacco leaves lose the capacity for *de novo* assembly of PSI (Krech *et al.*, 2012). This is also supported by the determination of very low daily turnover rates of PSI subunits in barley (*Hordeum vulgare*) leaves via *in planta* isotope labelling studies (Nelson *et al.*, 2014). Because our acclimation experiments were performed in young source leaves, which had just fully established their photosynthetic apparatus, it is possible that a decreased stability of PSI was compensated for by an increased *de novo* biogenesis of the complex. In senescing leaves, after PSI assembly has ceased, this might not be possible anymore, explaining the more pronounced loss of PSI there. A similar role in complex stability during leaf ontogenesis has previously been described for the small non-essential PetL subunit of the b_6_f (Schöttler *et al.*, 2007b). In its absence, b_6_f accumulation is unaltered in young leaves, but an accelerated loss of the b_6_f occurs in mature leaves. In these leaves, *de novo* biogenesis of the b_6_f has ceased, so that the lower stability of the mutant complex becomes apparent (Schöttler *et al.*, 2007b; Hojka *et al.*, 2014; Schöttler *et al.*, 2015).

Alternatively, the minor loss of PSI under high-light and chilling conditions could be attributable to different mechanisms than that during leaf senescence. Both chilling and high-light stress should result in an increased production of photosynthetic ROS. Indeed, the determination of thylakoid protein oxidation levels (Supplementary Fig. S5) showed that under all growth light regimes, but especially under high-light stress, thylakoid protein oxidation was increased in the mutants. An altered or impaired binding of one or more of the four carotenoids, which are normally coordinated by the I-subunit (Amunts *et al.*, 2010), could result in increased ROS production by PSI or impaired ROS detoxification.

On the other hand, senescing tobacco leaves are heavily shaded by younger leaves, resulting in strongly light-limited photosynthesis. This means that photosynthetic ROS production should be low, and a possible impairment of ROS detoxification should be irrelevant. Therefore, a scenario based on increased oxidative stress in the ∆*psaI* transformants is unlikely to explain the more pronounced loss of PSI in ageing leaves. Instead, during senescence, PSI might be more prone to proteolytic degradation either by a specific protease involved in its breakdown, or by a protease, which does not recognize wild-type PSI as a substrate. Because a large number of proteases are induced or upregulated during leaf senescence (reviewed by Roberts *et al.*, 2012), such a critical protease might not be present during the earlier developmental phases of the leaf. Unfortunately, not much is known about proteases involved in the degradation of PSI in higher plants. The degradation of PSI from *Chlamydomonas reinhardtii* was only studied *in vitro* after the addition of soluble proteases (Henderson *et al.*, 2003), but it is unclear to what extent these results can be extrapolated to the *in situ* degradation of PSI in intact leaves of higher plants. A role for the FTSH protease in PSI biogenesis and accumulation was recently described; however, this effect was unrelated to PSI turnover and degradation (Järvi *et al.*, 2016). In conclusion, in comparison to the general defect in PSI accumulation observed here under different environmental conditions and during leaf senescence, the stabilization of the LHCII docking site of PSI likely is a minor function of the I-subunit.

### Antenna structure

As already discussed above, the I-subunit is not relevant for state transitions. Several arguments also suggest that it is not required for efficient light harvesting and exciton distribution between the two photosystems. We did not observe a growth phenotype under low-light conditions (Fig. 2), when any defect in light harvesting and excitation energy distribution between the photosystems should have directly affected growth. The accumulation of LHCI proteins was unaltered (Fig. 5), and all LHCI proteins were efficiently coupled to the PSI reaction centre (Fig. 6). Whenever uncoupled LHCI accumulate, additional bands appear between wavelengths of 705 and 730 nm in 77 K chlorophyll-a fluorescence emission spectra. This was not the case in the ∆*psaI* mutants, but has been seen in many mutants suffering either from reductions in PSI accumulation (Albus *et al.*, 2010; Krech *et al.*, 2012) or from disturbances in the LHCI binding sites, such as the ∆*psaJ* mutant of tobacco (Schöttler *et al.*, 2007a). Normal binding of the LHCI in the ∆*psaI* knockout plants is unsurprising, because the I-subunit is located on the opposite side of PSI (Amunts *et al.*, 2010).

Furthermore, the unaltered light-response curves of qL (a measure for the redox state of the PSII acceptor side; Fig. 7D–F), and of the donor-side limitation of PSI (Fig. 8A–C) argue against any disturbance at the level of PSI excitation. Any compromised function of PSI would result in an imbalance in the excitation rates of PSII and PSI and a more reduced steady-state redox state of the PSII acceptor side (i.e. lower qL values). This was, however, not the case (Fig. 7). Likewise, the PSI donor-side limitation is a measure for the amount of photo-oxidized P_700_^+^, whose reduction is limited by electron transfer from PSII via the high-potential chain towards PSI. Under light-limited conditions, an impaired PSI antenna function would result in a lower rate of P_700_ photo-oxidation and less pronounced donor-side limitation. This effect was not observed in the ∆*psaI* plants (Fig. 8A–C). Most importantly, the quantum efficiency of CO_2_ fixation did not differ between the wild type and the transplastomic lines. Taking all these data together, we exclude a role for the I-subunit in light harvesting of PSI.

### The PSI I-subunit is not involved in redox reactions of PSI with plastocyanin or ferredoxin

The I-subunit is required for neither the redox reactions of PSI with plastocyanin or with ferredoxin. The identical capacities of linear electron flux (Fig. 3E) and leaf assimilation (Table 1) argue against a major problem in these reactions. Additionally, we directly determined the interaction of plastocyanin with PSI by measuring redox equilibration kinetics during the rapid reduction of both components during a short interval of darkness (Supplementary Fig. S2). No differences were observed between the wild type and the transplastomic lines. An impaired forward electron transfer towards ferredoxin and NADP^+^ would result in an accelerated acceptor-side limitation of PSI, which we did not observe under any growth condition (Fig. 8). Also, impaired ferredoxin reduction massively increases ROS production at the PSI acceptor side. *A. thaliana* mutants deficient in PSAD (Haldrup *et al.*, 2003) and PSAE (Varotto *et al.*, 2000), which are both required for correct formation of the stromal ridge and for ferredoxin reduction, are highly sensitive to photoinhibition even in dim light. We did not observe any such behaviour in our ∆*psaI* plants.

### Evolutionary aspects

Following endosymbiosis, the structure of PSI structure has undergone severe changes. In cyanobacteria, PSI is organized in the form of trimers, whereas in higher plants it functions as a monomer, binding newly evolved membrane-intrinsic LHC proteins. This led to a complete remodelling of the PSI surfaces within the thylakoid membrane. While the neighbouring subunit of PsaI in cyanobacterial PSI, PsaM, was lost during the evolution of eukaryotic PSI (which instead acquired the new subunit PSAH), the I-subunit was retained in the plastid genome. Among all plastid-encoded PSI subunits, *psaI*, together with *psaJ*, shows the lowest level of sequence similarity between cyanobacteria and higher plants, and also within higher plants (Yu *et al.*, 2008). PsaA, PsaB, and PsaC are much more highly conserved, likely because they bind the redox-active cofactors and constitute the PSI reaction centre. The legume genus *Lathyrus* even lost *psaI* due to complex plastome rearrangements (Magee *et al.*, 2010). A low selective pressure to maintain the I-subunit is in line with the fact that we only observed a small growth defect in our ∆*psaI* transformants under high-light conditions, while no phenotype was apparent under all other tested conditions. The accelerated loss of PSI in senescing leaves is unlikely to be a major selective force in those plant species in which the senescing leaves are largely shaded by younger leaves, so that their contribution to total plant assimilation is low. Whether there is a correlation between plant architecture and the functional importance of PsaI will be interesting to investigate.

## Supplementary Material

Supplementary Data

## Abbreviations:

b_6_f: cytochrome b_6_f complex
LHC: light harvesting complex
PSI: photosystem I
PSII: photosystem II
pmf: proton motive force
ROS: reactive oxygen species
SDG: sucrose density gradient.
