# Supplementary material for: The plastid-encoded PsaI subunit stabilizes photosystem I during leaf senescence in tobacco
Source: J Exp Bot. 2017 Feb 9;68(5):1137–55. doi: 10.1093/jxb/erx009 (PMC5429015; doi:10.1093/jxb/erx009)

Supplementary Material for:

The plastid-encoded PsaI subunit stabilizes photosystem I during leaf senescence in tobacco

Author names:

Mark Aurel Schöttler[1,2], Wolfram Thiele[1], Karolina Belkius[1], Sonja Verena Bergner[1], Claudia Flügel[1], Gal Wittenberg[1], Shreya Agrawal[1], Sandra Stegemann[1], Stephanie Ruf[1], and Ralph Bock[1]

[1]Max Planck Institute of Molecular Plant Physiology, Am Mühlenberg 1, 14476 Potsdam-Golm, Germany

[2]Corresponding author: Dr. Mark Aurel Schöttler, Max Planck Institute of Molecular Plant Physiology, Am Mühlenberg 1, 14476 Potsdam-Golm, Germany, Telephone: +49-331-5678311, E-mail: Schoettler@mpimp-golm.mpg.de

Supplementary Figure S1: Antenna functions of wild-type tobacco and the Δ*psaI* mutants grown at 350 µE m^{-2} s^{-1} light intensity. (A) Light response curves of leaf gas exchange were measured under CO_2-saturated conditions (2000 ppm CO_2), to determine leaf respiration in darkness, the light-saturated assimilation capacity, and the quantum efficiency of CO_2 fixation. (B) The quantum efficiency of CO_2 fixation was calculated from the slope of the light response curve under light-limited conditions between zero and 100 µE m^{-2} s^{-1}. Both the wild type and the transplastomic lines needed approximately 12 quanta per assimilated molecule of CO_2. (C) State Transitions were assessed by exposing plants to low intensity actinic illumination enriched in blue light, to preferentially excite PSII and induce a transition to state 2, and far-red light, to preferentially excite PSI and induce a transition to state 1. Exemplary chlorophyll a fluorescence kinetics of state transitions are shown for the wild type and the two Δ*psaI* mutants. Plants were dark-adapted for 30 min, followed by the determination of F_0 (normalized to one) and F_M. Then, blue-enriched PSII light was applied for 15 min, followed by 15 min of far-red enriched PSI light. This light treatment was repeated immediately afterwards.

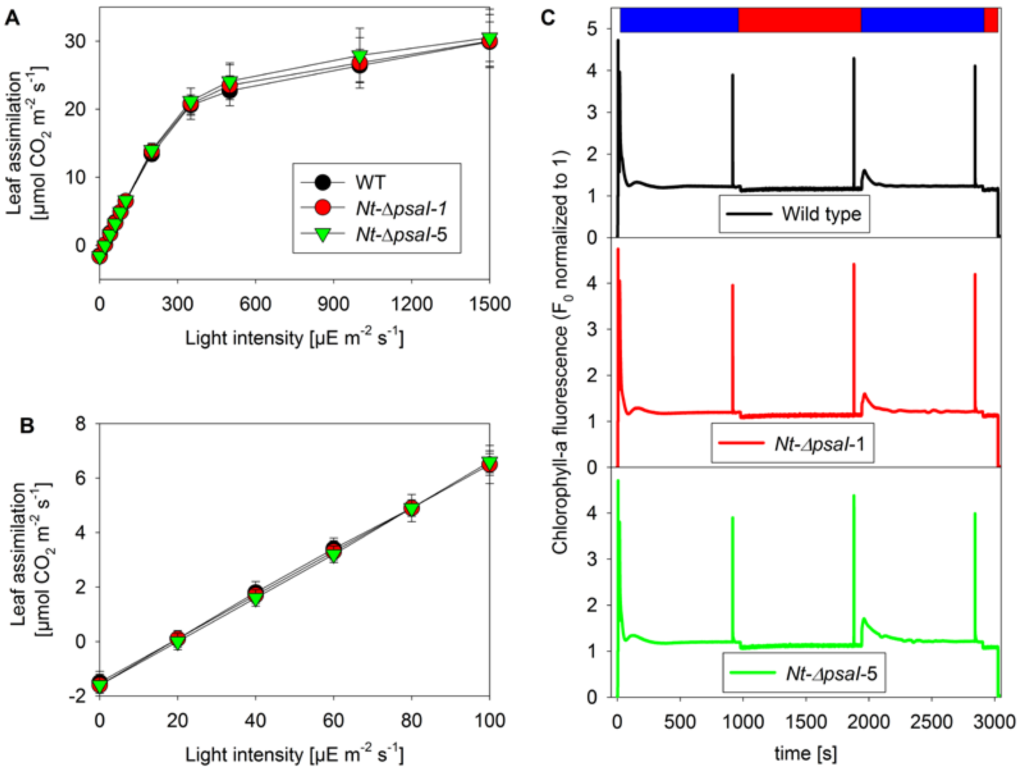

Supplementary Figure S2: Redox equilibration between plastocyanin and PSI. (A) Exemplary reduction kinetics of photo-oxidized plastocyanin and P_{700} from tobacco wild-type leaves. Intact leaves were illuminated with saturating light, to fully oxidize both plastocyanin and P_{700}, followed by a short interval of darkness, to allow the complete reduction of the high potential chain. The fully oxidized states of plastocyanin and P_{700}^{+} were normalized to 1, their fully reduced states were normalized to 0. (B) Redox equilibration plot of P_{700} (x-axis) and plastocyanin (y-axis). Again, the fully oxidized state of plastocyanin and P_{700}^{+} at the end of the saturating light pulse was normalized to 1, the reduced state in darkness to 0. The original redox equilibration curve (data, black symbols) was fitted with a mathematical model (fit, red symbols) to determine an apparent redox equilibration constant (K_{app}).

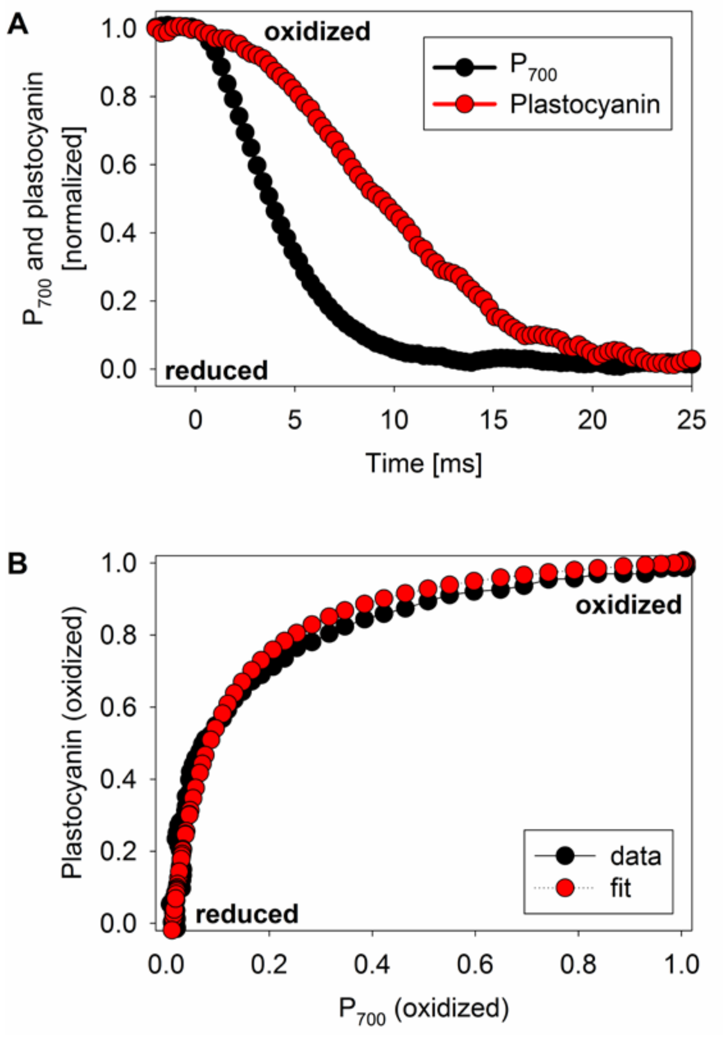

Supplementary Figure S3: Acclimation kinetic of mature leaves pre-adapted to low-light conditions (100 µE m^{-2} s^{-1}, 22°C day temperature) to a combination of intermediate light intensity (350 µE m^{-2} s^{-1}) and chilling stress (12°C during the day, 8°C during the night). Changes in leaf length (A), chlorophyll a/b ratio (B), chlorophyll content (C), maximum quantum efficiency of PSII in the dark-adapted state (F_V/F_M; D), and linear electron flux (E) were followed for 14 days. The stars indicate significant differences between wild type and mutants.

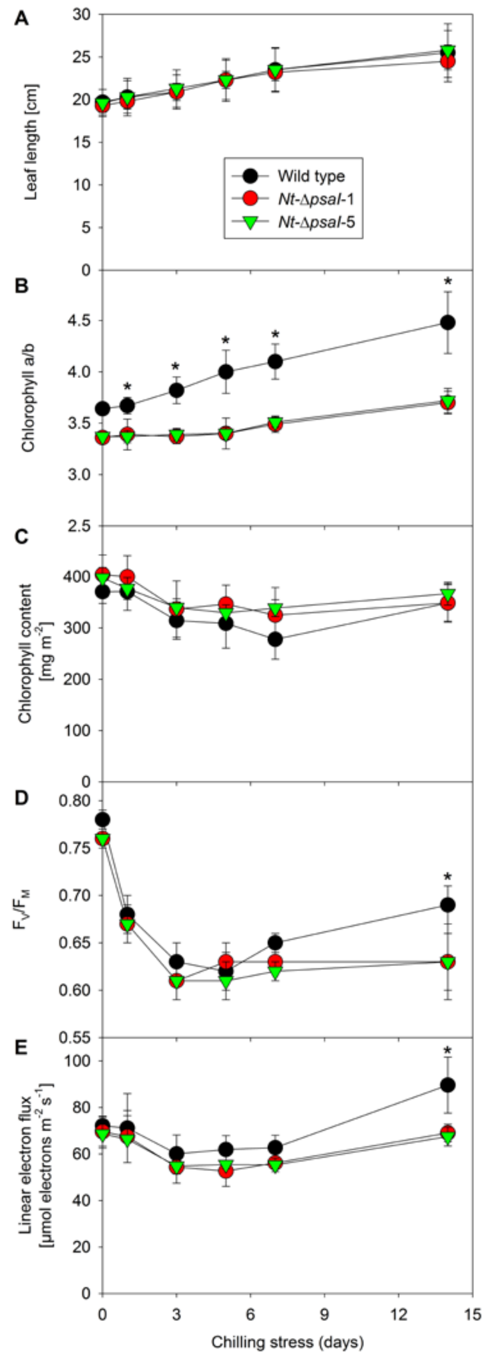

Supplementary Figure S4: Sucrose density gradient separation and immunoblot analyses of thylakoid protein complexes from wild-type tobacco and the two transplastomic Δ*psaI* lines. Separation of photosynthetic complexes was obtained by SDG centrifugation after solubilization of isolated thylakoids with DDM. Subsequently, SDGs (shown at the right side of the figure) were fractionated from bottom to top and an equal volume of the fractions five to 30 was subjected to immunodetection. As diagnostic subunit for PSII, PsbD (D2) was used, and the distribution of PSII inner antenna proteins was followed with an antibody against LHCB4. For PSI, in addition to the essential subunit PsaA, distribution of PSAL and PSAH was determined. Deletion of PsaI did not alter the distribution and association of the two nuclear-encoded subunits.

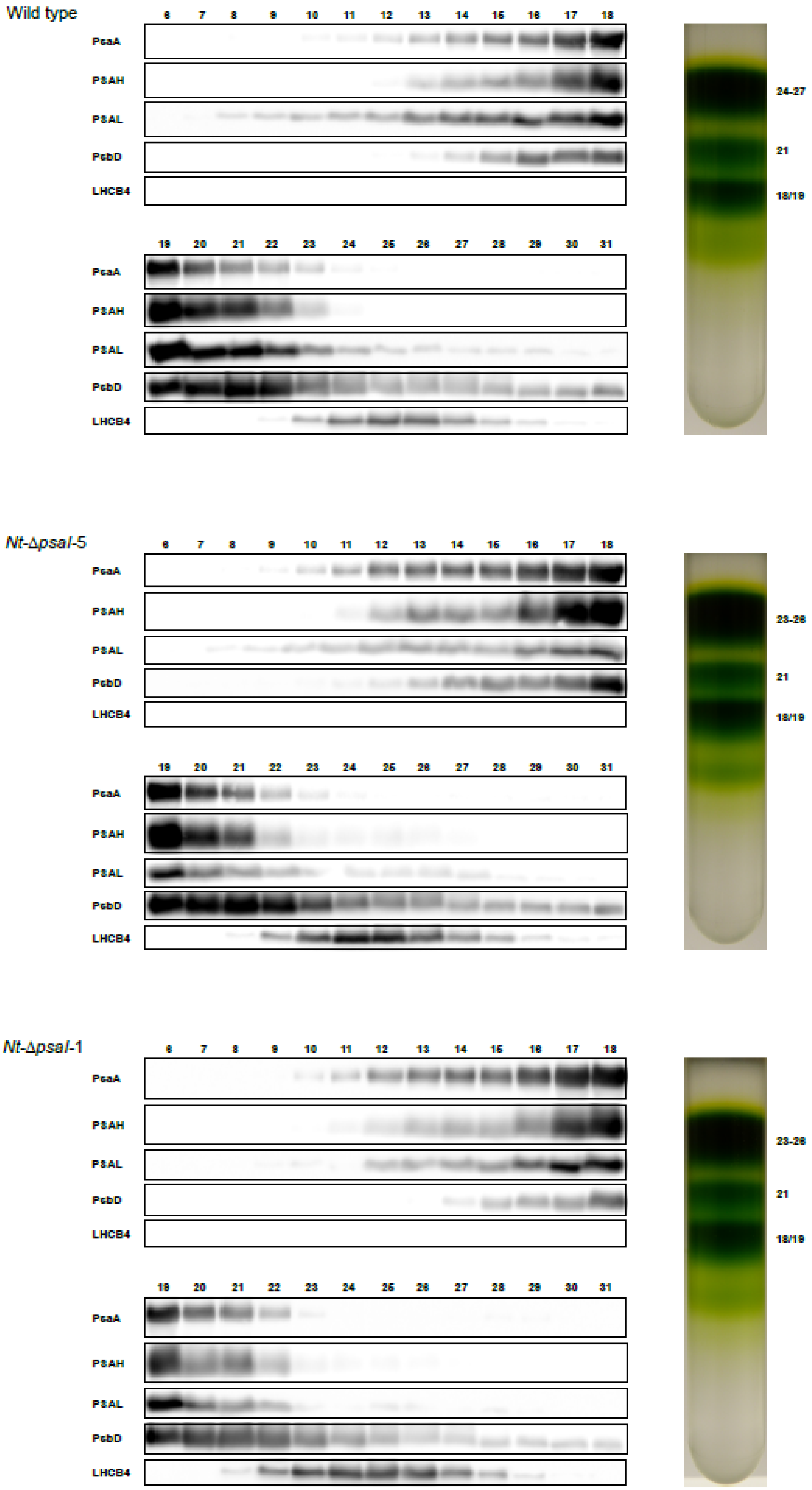
Wild type
6 7 8 9 10 11 12 13 14 15 16 17 18
PsaA
PSAH
PSAL
PsbD
LHCB4
19 20 21 22 23 24 25 26 27 28 29 30 31
PsaA
PSAH
PSAL
PsbD
LHCB4
24-27
21
18/19
Nt-Δpsal-5
6 7 8 9 10 11 12 13 14 15 16 17 18
PsaA
PSAH
PSAL
PsbD
LHCB4
19 20 21 22 23 24 25 26 27 28 29 30 31
PsaA
PSAH
PSAL
PsbD
LHCB4
23-26
21
18/19
Nt-Δpsal-1
6 7 8 9 10 11 12 13 14 15 16 17 18
PsaA
PSAH
PSAL
PsbD
LHCB4
19 20 21 22 23 24 25 26 27 28 29 30 31
PsaA
PSAH
PSAL
PsbD
LHCB4
23-26
21
18/19

Supplementary Figure S5: Oxidation of thylakoid proteins in wild type and mutants grown under low, intermediate and high-light conditions was determined using the Oxyblot protein oxidation detection kit. Lanes one to three contain wild type and the two transplastomic lines grown at 100 µE m^{-2} s^{-1}. Lanes four to six contain wild-type tobacco and the mutants grown at intermediate light intensities, and lanes seven to nine contain samples grown at high light intensities. Thylakoid proteins equivalent to 2 µg chlorophyll were loaded per reaction.

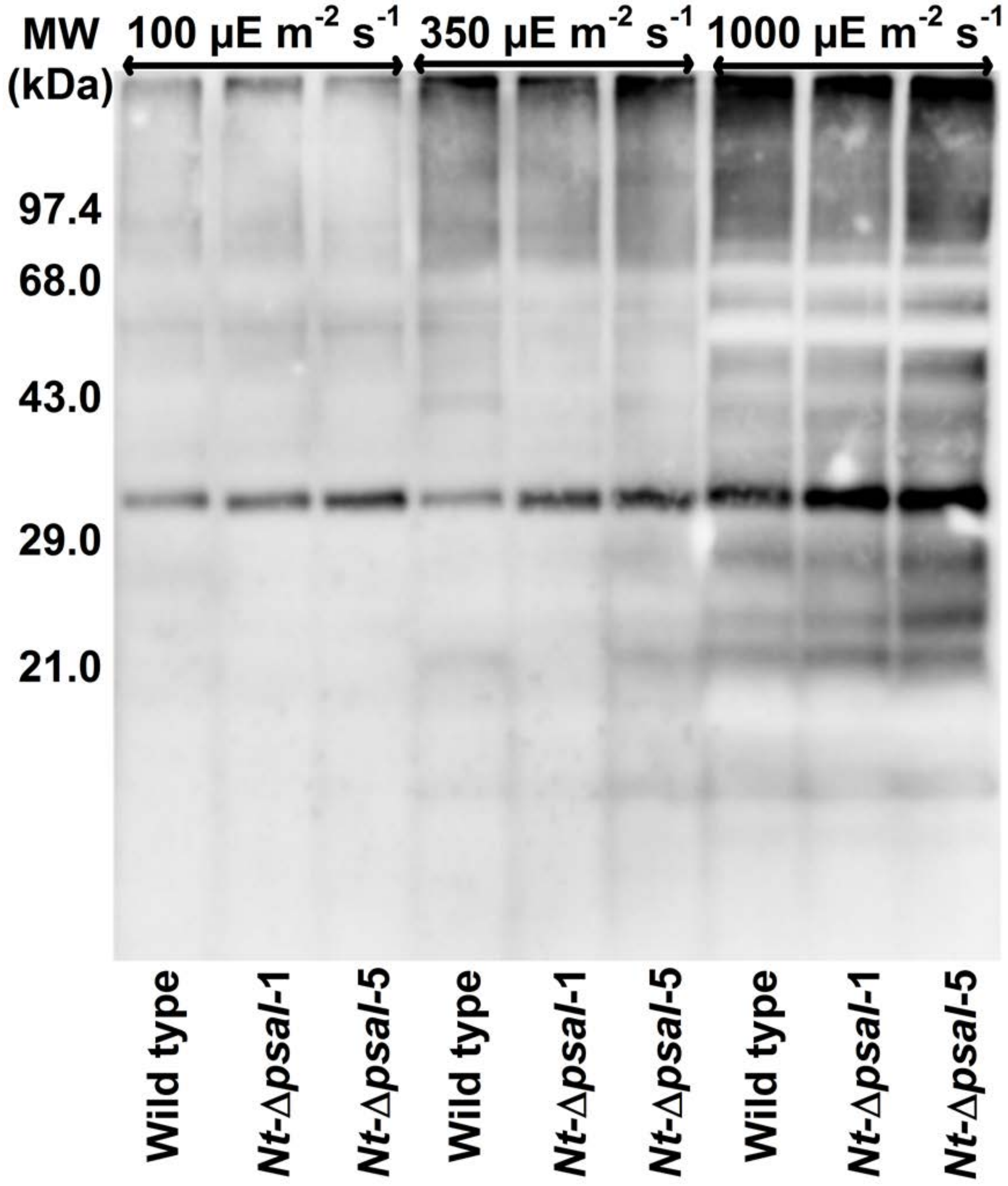

Supplement: Supplementary Data [file erx009_Supplementary_Data.zip › supplementary_figures_S1_S5.pdf]
